# Measuring maternal line selection driven by differential survival in ex situ collections for plant conservation

**DOI:** 10.1111/cobi.70350

**Published:** 2026-07-03

**Authors:** Rebecca Sucher, Taylor AuBuchon‐Elder, Georgia Thomas, Maura Collins, Allison Rea, Lauren Martin, Iván Jiménez

**Affiliations:** ^1^ Horticulture Division Missouri Botanical Garden St. Louis Missouri USA; ^2^ Division of Natural Sciences and Mathematics Washington College Chestertown Maryland USA; ^3^ Department of Biology Washington University in St. Louis St. Louis Missouri USA; ^4^ Department of Physical and Environmental Sciences Colorado Mesa University Grand Junction Colorado USA; ^5^ Center for Conservation and Sustainable Development, Science and Conservation Division Missouri Botanical Garden St. Louis Missouri USA

**Keywords:** attrition, demographic stochasticity, ex situ conservation, living plant collections, opportunity for selection, provenance selection, survival, Colecciones de plantas vivas, conservación ex situ, estocasticidad demográfica, desgaste, oportunidad de selección, selección por procedencia, supervivencia

## Abstract

Ex situ plant collections may be increasingly needed to protect representative samples of threatened or rare species and provide plant material for conservation translocation. A primary problem in these ex situ collections is loss of intraspecific variation due to plant death. Different management actions would be required depending on whether such loss results from demographic stochasticity or selection. We developed a method to test for maternal line selection according to provenance, given that maternal line and provenance serve as convenient and important partitions of the intraspecific variation represented in ex situ plant collections. The method tests for provenance selection (i.e., the differential survival of plants in maternal lines sourced from different geographic regions) and opportunity for selection within provenances (i.e., differential survival among plants in maternal lines sourced from the same geographic region). We used data from an ex situ collection of 243 saplings sourced from eight provenance regions and 42 maternal lines of Arkansas oak (*Quercus arkansana*), a vulnerable tree species restricted to small, scattered populations. We found no statistically significant provenance selection or opportunity for selection among the maternal lines during a 10.5‐month period, despite low overall survival rate (0.531) and large variation in survival rate among provenances (0.250–0.833) and maternal lines (0–1). By distinguishing survival patterns expected solely under demographic stochasticity from those likely resulting from selection, our method can be used to inform management of a wide range of ex situ collections for plant conservation.

## Abstract (es)

Medición de la selección de la línea materna impulsada por la supervivencia diferencial en colecciones ex situ para la conservación de plantas

**Resumen:**

Las colecciones de plantas ex situ pueden ser cada vez más necesarias para proteger muestras representativas de especies amenazadas o raras y proporcionar material vegetal para la translocación con fines de conservación. Un problema fundamental en estas colecciones ex situ es la pérdida de variación intraespecífica debido a la muerte de las plantas. Se requerirían diferentes medidas de manejo dependiendo de si dicha pérdida se debe a la estocasticidad demográfica o a la selección. Desarrollamos un método para evaluar la selección de líneas maternas según la procedencia, dado que la línea materna y la procedencia sirven como divisiones convenientes e importantes de la variación intraespecífica representada en las colecciones de plantas ex situ. El método evalúa la selección por procedencia (es decir, la supervivencia diferencial de las plantas en líneas maternas provenientes de diferentes regiones geográficas) y la oportunidad de selección dentro de las procedencias (es decir, la supervivencia diferencial entre plantas de líneas maternas provenientes de la misma región geográfica). Utilizamos datos de una colección ex situ de 243 plantines provenientes de ocho regiones de procedencia y 42 líneas maternas del roble de Arkansas (*Quercus arkansana*), una especie arbórea vulnerable restringida a poblaciones pequeñas y dispersas. No encontramos ninguna selección por procedencia ni oportunidad de selección estadísticamente significativas entre las líneas maternas durante un período de 10.5 meses, a pesar de la baja tasa de supervivencia general (0.531) y la gran variación en la tasa de supervivencia entre procedencias (0.250‐0.833) y líneas maternas (0‐1). Al distinguir los patrones de supervivencia esperados únicamente bajo la estocasticidad demográfica de aquellos que probablemente resulten de la selección, nuestro método puede utilizarse para orientar el manejo de una amplia gama de colecciones ex situ para la conservación de plantas.

## INTRODUCTION

As anthropogenic pressures threatening plant diversity grow (Nic Lughadha et al., 2020), biodiversity conservation strategies may need to rely more heavily on ex situ plant collections (Heywood, 2017; Volis, 2017), broadly understood as populations of species under intense management (IUCN/SSC, 2014). These ex situ collections include seed and spore banks, cryopreserved tissue collections, whole growing plant collections, and collections of living plant material that support in situ species conservation in multiple ways (IUCN/SSC, 2014), such as providing plant material for reinforcement or reintroduction programs (Westwood et al., 2021) and temporarily protecting a representative sample of a species from threats experienced by wild populations (Smith & Pence, 2017). Ex situ collections are often managed with the aim of representing a significant part of the variation within a species (Center for Plant Conservation, 2019). This entails collecting and maintaining living plant material from multiple maternal lines and provenances across the geographic range of a species (Griffith et al., 2021; Hoban, 2019; Hoban et al., 2020), a task that is often difficult (Wei & Jiang, 2021; but see Forgiarini et al., 2023) yet important for conservation success. The greater the number of maternal lines represented in a reinforcement or reintroduction program, the greater the intraspecific variation represented, thereby increasing the likelihood of establishing resilient, self‐sustaining populations in the wild (Center for Plant Conservation, 2019; Hoban et al., 2023). Collecting adequate samples across a species’ range may present logistical challenges, including accessibility constraints, resource limitations, and coordination across multiple sites with different land ownership. Moreover, even if collecting hurdles are overcome, the amount of intraspecific variation initially represented in an ex situ collection will inexorably diminish over time due to plant death, a process referred to as attrition (Guerrant & Fielder, 2004; Hoban, 2019; Thomas et al., 2023).

Attrition in ex situ collections due to plant death is often conceptualized based on a species‐level death rate parameter that may vary according to properties of ex situ collections (Hoban, 2019). For example, the seed storage conditions that minimize death rates can vary across species (Chau et al., 2019). Beyond species‐level death rates, quantifying intraspecific variation in death rates according to maternal line and provenance may help elucidate the mechanisms by which plant death erodes intraspecific variation in ex situ collections, thereby informing ex situ conservation programs (Thomas et al., 2022, 2023; van der Merwe et al., 2023). Focusing on maternal lines and provenance seems useful because ex situ plant collections are primarily built by collecting seeds or other propagules from individual mother plants that belong to different wild populations across the geographic range of a species. The seeds or propagules from each mother plant correspond to maternal lines that are ideally maintained as distinct accessions, along with information on provenance, in standard practices for the acquisition and curation of plant materials in ex situ collections for plant conservation (Center for Plant Conservation, 2019).

Maternal lines and provenance are emphasized in standard practices because they serve as convenient and important partitions of the intraspecific variation represented in ex situ plant collections. Maternal line information reflects a significant component of relatedness among individuals because commonly half the nuclear genome is maternally inherited and, in cases including apomixis and development of new individuals from somatic tissues (Bicknell & Koltunow, 2004; Hartman et al., 2010), the whole nuclear genome is maternally inherited. In many angiosperms, the cytoplasmic and plastid genomes are maternally inherited (Birky, 1995). Moreover, nongenetic maternal effects are common in plants (Roach & Wulff, 1987) and important for local adaptation (Herman & Sultan, 2011) and restoration programs (Espeland & Hammond, 2013). Thus, maternal line information is useful to gauge the amount of intraspecific variation represented in an ex situ collection (Hoban, 2019; van der Merwe et al., 2023). Similarly, provenance serves as a proxy variable for intraspecific geographic variation, including local adaptation, which is highly relevant for reinforcement and restoration programs (Baughman et al., 2019; Erickson & Halford, 2020).

Therefore, studying the mechanisms underpinning the distribution of plant deaths across maternal lines and provenances would seem to be a practical approach to identify management practices that ameliorate the loss of intraspecific variation through time due to attrition in ex situ collections. Selection, understood as consistent differences between phenotypes in relative fitness within a generation (Arnold & Wade, 1984; Frank, 2012), is commonly thought to diminish intraspecific variation via attrition at many steps in the management of ex situ collections (Havens et al., 2004; Ensslin & Godefroid, 2019; Rauschkolb et al., 2019). Unintentional or unconscious selection, arising from selective pressures imposed by human‐managed environments, without deliberate human intent to favor particular phenotypes (Walsh & Lynch, 2018; Zohary, 2004), might be common in ex situ collections (Ensslin & Godefroid, 2019; Havens et al., 2004). For example, seeds from particular maternal lines or provenances may not withstand ex situ storage conditions or the germination regime that follows (Galíndez et al., 2019; Nagel et al., 2015). Additionally, seedlings or 6 saplings from a subset of maternal lines or provenances may be unlikely to survive the conditions of a garden or greenhouse (Havens et al., 2004). In contrast, intentional or conscious selection occurs when humans intentionally select desirable traits in a population (Walsh & Lynch, 2018; Zohary, 2004). For example, plants from some maternal lines or provenances may be selected by horticulturists based on superior vigor or aesthetic qualities (Ensslin & Godefroid, 2019; Havens et al., 2004). Both unintentional and intentional selection in ex situ collections may favor plant phenotypes with low probability of survival or reproduction in the wild (Zohary, 2004), potentially compromising reinforcement or reintroduction programs (Ensslin & Godefroid, 2019; Havens et al., 2004; Rauschkolb et al., 2019). In principle, ex situ collections for plant conservation could be managed to eliminate or reduce unintentional and intentional selection, by varying the storage or cultivation environments according to maternal line or provenance, given the underlying differences in relative fitness are quantified and understood.

**FIGURE 1:**
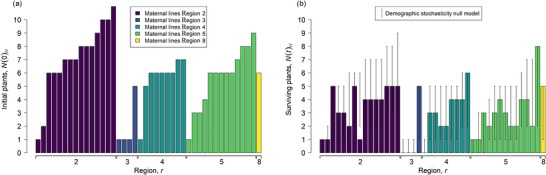
Distribution of *Quercus arkansana* saplings among maternal lines and provenance regions represented in an ex situ collection at the Oertli Family Hardy Plant Nursery of the Missouri Botanical Garden (a) in November 2022, when there were 243 saplings alive, and (b) 10.5 months later (by mid‐September 2023), when only 129 saplings remained alive (error bars, 95% confidence intervals for a null model of demographic stochasticity; *x*‐axis numbers, provenance regions from Thomas et al. [2023]).

However, not all variation in mortality rates among provenances and maternal lines in ex situ collections is necessarily due to selection. Plant death could be random with respect to provenance and maternal line, reflecting demographic stochasticity (Lande, 1993) and resulting in genetic drift (Lenormand et al., 2009). In fact, demographic stochasticity is often regarded as a null model in assessments of selection (Walsh & Lynch, 2018; Waples & Reed, 2023). Under this approach, demographic stochasticity is considered a simpler explanation because it only requires stochastic differences in relative fitness between phenotypes, which are bound to occur merely as a result of finite population size (Gotelli & McGill, 2006). Thus, evidence of selection entails observed differences in relative fitness between phenotypes that exceed in magnitude the differences that would be expected solely from demographic stochasticity. The loss of intraspecific variation through demographic stochasticity is a function of the size of the ex situ collection; larger losses are expected in smaller collections. This loss may be tallied in terms of the change over time in the frequency of individual plants from different provenances and maternal lines in the ex situ collection, as illustrated for an ex situ collection in Figure 1. If an ex situ collection is small and not supplemented with new collections from wild populations (Havens et al., 2004; Lacy, 2013), large changes in the relative frequency of provenances and maternal lines would be expected due solely to demographic stochasticity. Ex situ collections for plant conservation could be managed to reduce the effects of demographic stochasticity by equalizing the representation of different provenances and maternal lines (Havens et al., 2004). However, management to counter the effects of demographic stochasticity could entail different practices from those required to counter selection. Only in the case of management to counter selection would it make sense to vary storage or cultivation environments according to provenance or maternal line.

The management of ex situ collections may thus be informed by assessments of whether selection, unintentional or intentional, underpins the distribution of plant deaths among provenances and maternal lines and, ultimately, the decline in intraspecific variation through time commonly observed in plant ex situ collections (Wei & Jiang, 2021). However, we are unaware of approaches specifically designed to conduct such assessments based on null models of demographic stochasticity. We sought to fill this gap by devising methods to measure maternal line selection with broad applicability to ex situ collections for plant conservation. We also considered how these methods can inform the management of ex situ collections for plant conservation.

## CONCEPTUAL BASIS

Our approach to measuring maternal line selection due to differential survival in ex situ collections for conservation can be applied to ex situ collections that represent intraspecific variation in a focal species with maternal lines sourced from multiple geographic regions, multiple maternal lines per geographic region, and multiple individual plants per maternal line. These three conditions are not overly restrictive because they align with established best practices for ex situ plant conservation collections (Center for Plant Conservation, 2019). They can be met by ex situ collections that represent only maternal lines and provenances across part of a species’ geographic range. We assumed that the ex situ collection of the focal species is similar to a common garden experiment (Wadgymar et al., 2022), whereby environmental conditions are under considerable control and substantially standardized across individual plants. We considered how some aspects of the approach can be applied to ex situ collections that do not resemble a common garden experiment or meet only some of the three conditions above.

We based our approach on extensive previous work on measuring selection and opportunity for selection (Arnold & Wade, 1984; Reed et al., 2023; Walsh & Lynch, 2018). However, our approach differs from standard methods used to measure components of individual fitness, such as survival and reproduction. For example, individual fitness may be calculated based on survival to different ages and the respective fecundities at each age (Brommer, 2000; Lenski & Service, 1982; McGraw & Caswell, 1996). The survival of the offspring produced is generally not considered part of such calculations, with the possible exception of offspring survival during early ontogenetic stages because it mainly reflects offspring features rather than parental characteristics (Primack & Kang, 1989; Thomson & Hadfield, 2017; Wolf & Wade, 2001). In contrast, our approach focuses on the fitness of maternal lines in ex situ collections, as measured by the probability of survival of (full or half) siblings in each maternal line. This focus on maternal line fitness, as opposed to individual fitness, follows from the central role that management of maternal lines plays in representing and maintaining intraspecific variation in ex situ collections (Center for Plant Conservation, 2019; Havens et al., 2004). Similarly, the focus on survival as the sole measure of maternal line fitness, excluding reproduction, follows from the idea that ex situ collections for conservation would ideally maintain intraspecific variation through high survival rates and immigration via continued sampling efforts from wild populations and not by producing multiple generations ex situ (Havens et al., 2004). In short, this approach aims to measure maternal line fitness, based on the survival of siblings in ex situ collections, because it is highly relevant for the maintenance of intraspecific variation in ex situ collections.

We quantified observed patterns of attrition in an ex situ collection at two levels according to provenance: differential survival of plants in maternal lines sourced from different geographic areas (i.e., provenance selection) and differential survival among plants in maternal lines sourced from the same geographic area, measured as opportunity for selection (Arnold & Wade, 1984). Observed patterns were then compared with values generated by null models based on demographic stochasticity to test whether observed patterns are due to selection.

### Provenance selection

We defined provenance selection in an ex situ collection as differential survival among plants from maternal lines sourced from different locations within the geographic range of a focal species. Provenance selection is generally undesirable in ex situ collections that aim to represent intraspecific variation in species of conservation concern (Havens et al., 2004). Unfortunately, provenance selection may be widespread in ex situ collections because local adaptation is common in plants (Baughman et al., 2019; Lascoux et al., 2016; Leimu & Fischer, 2008; Wadgymar et al., 2022). Maternal lines sourced from different locations are likely endowed with different adaptive traits that cause differential survival in ex situ collections, as suggested by provenance trials (Ignazi et al., 2020; König, 2005; Morgenstern, 1996; Risk et al., 2021; Wright, 1976). To monitor provenance selection, we characterized the survival rate of plants in maternal lines sourced from different provenances as the following:

(1) $$\bar{S_{r}}\left(t\right)=\frac{\sum_{i=1}^{n_{r}}S_{ri}\left(t\right)}{n_{r}},$$

where $\bar{S_{r}}\left(t\right)$ is the mean survival rate of individual plants across maternal lines sourced from region r, $n_{r}$ is the number of maternal lines sourced from region r, and $S_{ri}\left(t\right)$ is the survival rate of plants from maternal line i sourced from region r. The $\bar{S_{r}}\left(t\right)$ and $S_{ri}\left(t\right)$ are measured over time span t:

(2) $$S_{ri}\left(t\right)=\frac{N_{ri}\left(t\right)}{N_{ri}\left(0\right)},$$

where $N_{ri}\left(0\right)$ and $N_{ri}\left(t\right)\;$ are the number of plants from maternal line i sourced from region r that were alive at the initial time and after time span t, respectively. Equation notation is summarized in Table 1.

**TABLE 1:** Summary of notation used in Equations (1) to (3).

Symbol	Definition
i	Maternal line
r	Provenance region
t	Time span over which survival is measured
$\bar{S_{r}}\left(t\right)$	Mean survival rate of individual plants across maternal lines sourced from region r measured over time t
$n_{r}$	Number of maternal lines sourced from region r
$S_{ri}\left(t\right)$	Survival rate of plants from maternal line i measured over time t
$N_{ri}\left(0\right)$	Number of plants from maternal line i sourced from region r that were alive at the onset of time interval *t*
$N_{ri}\left(t\right)$	Number of plants from maternal line i sourced from region r that were alive after time span t
$I_{r}\left(t\right)$	Opportunity for selection among maternal lines sourced from region r measured over time t

### Opportunity for selection

Opportunity for selection refers to variation in fitness among individuals. The larger such variation is, the larger is the maximum trait change that can take place within a generation due to selection (Arnold & Wade, 1984; Reed et al., 2023; Walsh & Lynch, 2018). Opportunity for selection is thus useful to monitor the potential for selection due to differential survival in ex situ collections. It is formally defined as the variance in relative fitness among individuals, equivalent to the squared coefficient of variation in absolute fitness. Accordingly, to monitor the potential for differential survival among maternal lines sourced from the same provenance, we defined opportunity for selection within a given region r, $I_{r}\left(t\right)$, as

(3) $$I_{r}\left(t\right)=\frac{\sum_{i=1}^{n_{r}}{\left(\frac{S_{\mathrm{ri}}\left(t\right)}{\overset{-}{S_{r}}\left(t\right)}\;-\;1\right)}^{2}}{n_{r}-1},$$

where $I_{r}\left(t\right)$ is the variance in relative fitness among maternal lines sourced from region r, whereby relative fitness is measured in terms of survival, $\frac{S_{ri}\left(t\right)}{\bar{S_{r}}\left(t\right)}$. This is equivalent to the squared coefficient of variation in absolute fitness among maternal lines from region r, where absolute fitness is $S_{ri}\left(t\right)$.

### Demographic stochasticity null models

We adopted the idea that demographic stochasticity serves as a null model in assessments of selection and opportunity for selection (Gotelli & McGill, 2006; Walsh & Lynch, 2018; Waples & Reed, 2023). Our approach tests whether observed differences in maternal line survival, among and within provenance regions in an ex situ collection, exceed what would be expected by demographic stochasticity. If they do, then provenance selection or large opportunity for selection due to differential survival would be inferred. We used two null models, one for provenance selection, as quantified by $\bar{S_{r}}\left(t\right)$, and another for opportunity for selection within regions, $I_{r}\left(t\right)$.

The null model for provenance selection randomly distributes individual plant deaths among maternal lines (i), irrespective of provenance (Figure 1). It preserves the number of individual plant deaths observed across the whole ex situ collection over time t but not their distribution among maternal lines (i) and provenance regions (r). At each iteration of this null model, a null value of $\bar{S_{r}}\left(t\right)$ is calculated on the randomized survival data. Multiple iterations produce a null distribution of $\bar{S_{r}}\left(t\right)$ that describes how likely different $\bar{S_{r}}\left(t\right)$ values would be if plant deaths across maternal lines were random with respect to provenance, as expected from demographic stochasticity alone, in the absence of provenance selection. Thus, observed values of $\bar{S_{r}}\left(t\right)$ in or beyond the extreme upper tail of this null distribution would be implausible if only demographic stochasticity, in the absence of provenance selection, were responsible for the distribution of plant deaths across maternal lines and provenances in an ex situ collection. Following convention, the extreme upper tail of a null distribution can be defined as the part of the distribution containing only the highest 1–5% values (Murtaugh, 2014). Accordingly, provenance selection would be inferred when an observed $\bar{S_{r}}\left(t\right)$ value lies beyond the lower limit of the upper 1–5% of the respective null distribution. The proportion of a null distribution lying beyond the observed value is the *p* value. A low *p* value (conventionally <0.05 [Murtaugh, 2014]) would alert managers to the likely occurrence of a generally undesirable selection process in an ex situ collection for conservation.

The null model for opportunity for selection within regions, $I_{r}\left(t\right)$, randomly distributes individual plant deaths among maternal lines (i) within a provenance region (r). It preserves the number of individual plant deaths observed across the whole ex situ collection and in each provenance region (r) over time t. At each iteration of this null model, a null value of $I_{r}\left(t\right)$ is calculated on the randomized survival data to construct a null distribution of $I_{r}\left(t\right)$ over multiple iterations. This null distribution describes how likely different $I_{r}\left(t\right)$ values would be if plant deaths were randomly distributed across maternal lines sourced from a provenance region, as expected from demographic stochasticity alone, in the absence of maternal line selection. Large opportunity for selection among maternal lines sourced from a region would be inferred when an observed $I_{r}\left(t\right)$ value lies beyond the lower limit of the upper tail of the respective null distribution, defined by the 1–5% highest null values (see above). Such a result would be unlikely if demographic stochasticity alone, in the absence of maternal line selection, were responsible for the distribution of plant deaths across maternal lines sourced from a particular provenance region. That result, documented by a *p* value indicating the proportion of the null distribution lying beyond the observed $I_{r}\left(t\right)$ value, would be generally undesirable in ex situ collections for conservation because it would indicate large potential for maternal line selection. Properties of the sampling distribution of $\bar{S_{r}}\left(t\right)$ and $I_{r}\left(t\right)$ under the demographic stochasticity null model are described in Appendix S1.

## METHODS

### Empirical example

**FIGURE 2:**
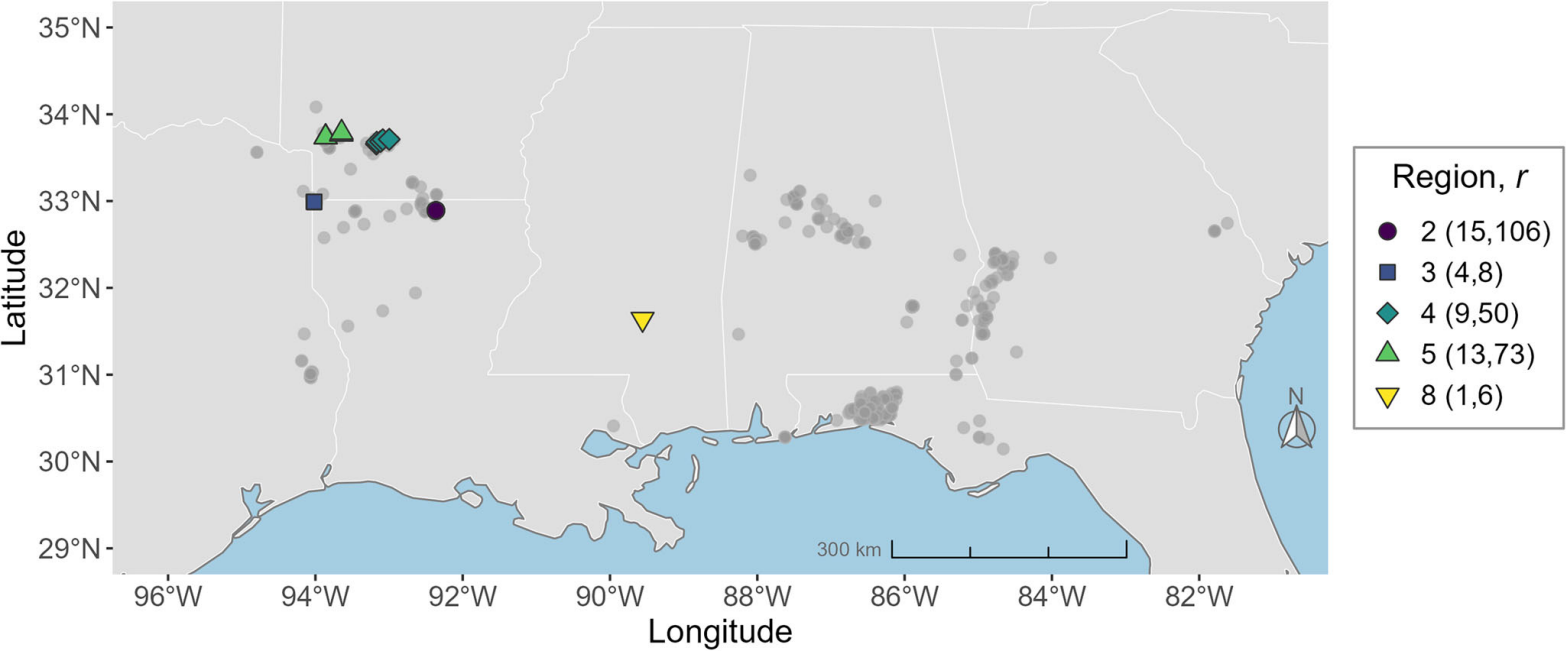
Known *Quercus arkansana* occurrences (GBIF.org) and location of maternal trees whose progenies were cultivated in an ex situ collection at the Oertli Family Hardy Plant Nursery of the Missouri Botanical Garden (dark circles and other symbols). The symbols show the provenance region of maternal trees according to the legend on the right; the two numbers in parentheses represent the number of maternal lines and the total number of initial plants per region, respectively. Regions 4 and 5, completely in Arkansas; Regions 2 and 3, between Arkansas and Louisiana; Region 8, in Mississippi; region numbers (2, 3, 4, 5, and 8) follow Thomas et al. (2023).

Here, we illustrate how practitioners may apply our approach to measure selection and opportunity for selection due to differential survival of maternal lines in an ex situ collection. We used data from an ex situ collection of *Quercus arkansana* saplings cultivated in the Oertli Family Hardy Plant Nursery at the Missouri Botanical Garden (MBG). This collection included multiple saplings from multiple maternal lines (wild trees) and populations across the species range (Figure 2).

### Study system

Arkansas oak (*Q. arkansana*) is a small, scrubby tree (15–40 feet tall) restricted to small, scattered populations in the southeastern United States. It occurs in shaded habitats of mixed scrub forest on ridges and bluffs along small waterways and prefers well‐drained soil. This species is considered critically imperiled in Texas; imperiled in Georgia and Louisiana; vulnerable in Arkansas, Alabama, and Florida; and vulnerable globally (NatureServe, 2025). The most secure habitats for remaining, fragmented populations tend to be steep head ravines. The main threats to *Q*. *arkansana* are commercial forestry practices, destruction of habitat for residential and commercial use, management of habitat to restore other species, and increasing drought associated with climate change (Beckman et al., 2019). Additional potential threats, noted during MBG‐led scouting and collecting trips in 2017 and 2021, include shot hole fungus, introgression, low seed set, and limited protection of populations in private lands.

In 2021, MBG staff and collaborators collected 2481 acorns from 48 wild trees in the western part of the species range (Figure 2). A unique accession number assigned to all acorns from a maternal tree was entered in MBG's Living Collections Management System (LCMS) (Missouri Botanical Garden, 2024), with detailed provenance and collection data for each maternal tree. The acorns were cold stratified for 134 days from November 2021 to March 2022. In March 2022, all acorns were sown in 10‐cm pots with Ball perennial mix substrate and placed in protected tree boxes outdoors. By early May 2022, 243 acorns had germinated and were transplanted into 3‐L air pots from August 2022 to November 2022 and moved to a hoop house where the saplings remained for the rest of the study period.

These saplings were part of a common garden experiment designed to assess intraspecific variation across maternal lines and geographic provenances under fairly uniform environmental conditions and standardized horticultural care. Each sapling was labeled with a unique tag including the accession, planting number, and a random number that determined their sequential position in the hoop house. This stochastic arrangement of saplings aimed to randomize, across maternal lines and provenances, potential effects of environmental heterogeneity within the hoop house related to environmental variables such as light and temperature. Although plants were occasionally moved within the hoop house and therefore did not stay in the exact same location throughout the study, their relative position remained consistent based on the preassigned random number. In the hoop house, the saplings were watered using overhead and hand‐watering methods and weeded as needed. By July 2023, the mean length of the main sapling stems reached 264.5 mm (SD 142.3), and the mean stem diameter at the base was 4.5 mm (SD 2.2). The sapling's pots were fertilized with Osmocote Plus 15‐9‐12, a slow‐release formula composed of 15% nitrogen, 9% phosphate, and 12% potash, plus secondary nutrients magnesium and sulfur and minor nutrients boron, copper, iron, manganese, molybdenum, and zinc.

### Data collection and analyses

Survival data for each of the 243 saplings were collected during two surveys. In the first survey, between 25 July and 3 August 2023, saplings were recorded as alive whenever they had green tissue, visible to the naked eye, on the buds, leaves, or stems. Saplings not meeting this criterion were further examined by scraping a small section of the bark with a clean tool and were recorded as alive if the exposed cambium was wet with a greenish hue. Otherwise, the sapling was recorded as possibly dead. There were 107 plantings categorized as possibly dead in the first survey. In the second survey on 19 September 2023, the same procedure was repeated. To confirm that plants categorized as possibly dead were indeed dead, we cut across the stems to ensure there was no sign of wet cambium or green tissue. All plants classified as possibly dead had dry, brittle, and brown stems and were thus recorded as dead. There were 114 plants confirmed dead in the second survey (Figure 1), including all 107 plants recorded as possibly dead in the first survey.

We used these survival data to measure provenance selection, based on Equation (1), and opportunity for selection within provenances based on Equation (3). We used the analysis in Thomas et al. (2023) to assign maternal trees to regions separated by at least 50 km, an approximation of the distance between distinct populations based on pollen dispersal distance in oaks (Beckman et al., 2019). This assignment resulted in five regions in Arkansas, Louisiana, and Mississippi (Figure 2), each with six to 106 individual saplings from one to 15 maternal trees (Figure 1).

We used R (R Core Team, 2024) to calculate provenance selection and opportunity for selection and to implement the respective null models. Because we performed several comparisons between observed and null distributions, we implemented the Holm–Bonferroni adjustment of *p* values to control the false discovery rate in statistical tests of multiple null hypotheses (Holm, 1979). To examine whether the location of the plants in the hoop house had a significant effect on survival, we conducted an analysis of spatial autocorrelation in individual plant survival and in the residuals of the logistic regression of individual plant survival on maternal line (Appendix S2). Annotated code is available at https://github.com/mobot‐living‐collections/ex‐situ‐selection.

## RESULTS

### Provenance selection

**FIGURE 3:**
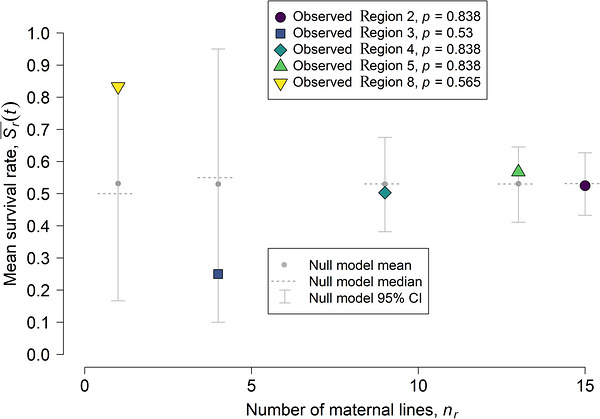
Observed mean survival rates for maternal lines of *Quercus arkansana* saplings sourced from five regions, after 10.5 months in an ex situ collection at the Oertli Family Hardy Plant Nursery of the Missouri Botanical Garden, and the respective distribution of survival rates according to the null model of demographic stochasticity (large symbols, mean survival rate estimated with Equation 1 for each provenance region; small points, null model mean; dotted lines, median null values; error bars, 95% confidence intervals of the null model; region numbers follow Thomas et al. [2023]).

Between November 2022, when the 243 saplings were placed in the nursery hoop house, and the last survey on 19 September 2023, the observed overall survival rate in the ex situ collection of *Q*. *arkansana* was 0.531 (129 saplings survived). The observed mean survival rates across maternal lines varied substantially among regions, between 0.250 (Region 3) and 0.833 (Region 8). However, this variation was well within the 95% confidence intervals of the demographic stochasticity null model for $\bar{S_{r}}\left(t\right)$ (Figure 3). The confidence intervals for $\bar{S_{r}}\left(t\right)$ were widest for Regions 3 (0.1–0.95) and 8 (0.167–0.833) because these two regions were represented by the lowest number of maternal lines ($n_{r}$) and initial number of individuals per maternal line, $N_{ri}\left(0\right)$, in the ex situ collection (Table 2; Figure 1). These results illustrate the potential for demographic stochasticity to generate relatively large variation in survival rates among provenances that are poorly represented in an ex situ collection in terms of $n_{r}$ or $N_{ri}\left(0\right)$ (Appendix S1). Such provenances may seem to show unusually low or high survival rates (e.g., Regions 3 and 8, respectively) that are nonetheless expected by purely stochastic processes (Figure 3). We found no evidence of provenance selection, as measured by differential survival rates among maternal lines sourced from different regions.

**TABLE 2:** Number of maternal lines and number of saplings alive at the initial time and after 10.5 months according to provenance region in an ex situ collection of *Quercus arkansana* at the Oertli Family Hardy Plant Nursery of the Missouri Botanical Garden.

Variable	Region 2	Region 3	Region 4	Region 5	Region 8
Maternal lines, $n_{r}$	15	4	9	13	1
Plants alive at the initial time, $\sum_{i=1}^{n_{r}}N_{ri}\left(0\right)$	106	8	50	73	6
Plants alive after *t* = 10.5 months, $\;\sum_{i=1}^{n_{r}}N_{ri}\left(t\right)$	52	5	28	39	5

### Opportunity for selection

**FIGURE 4:**
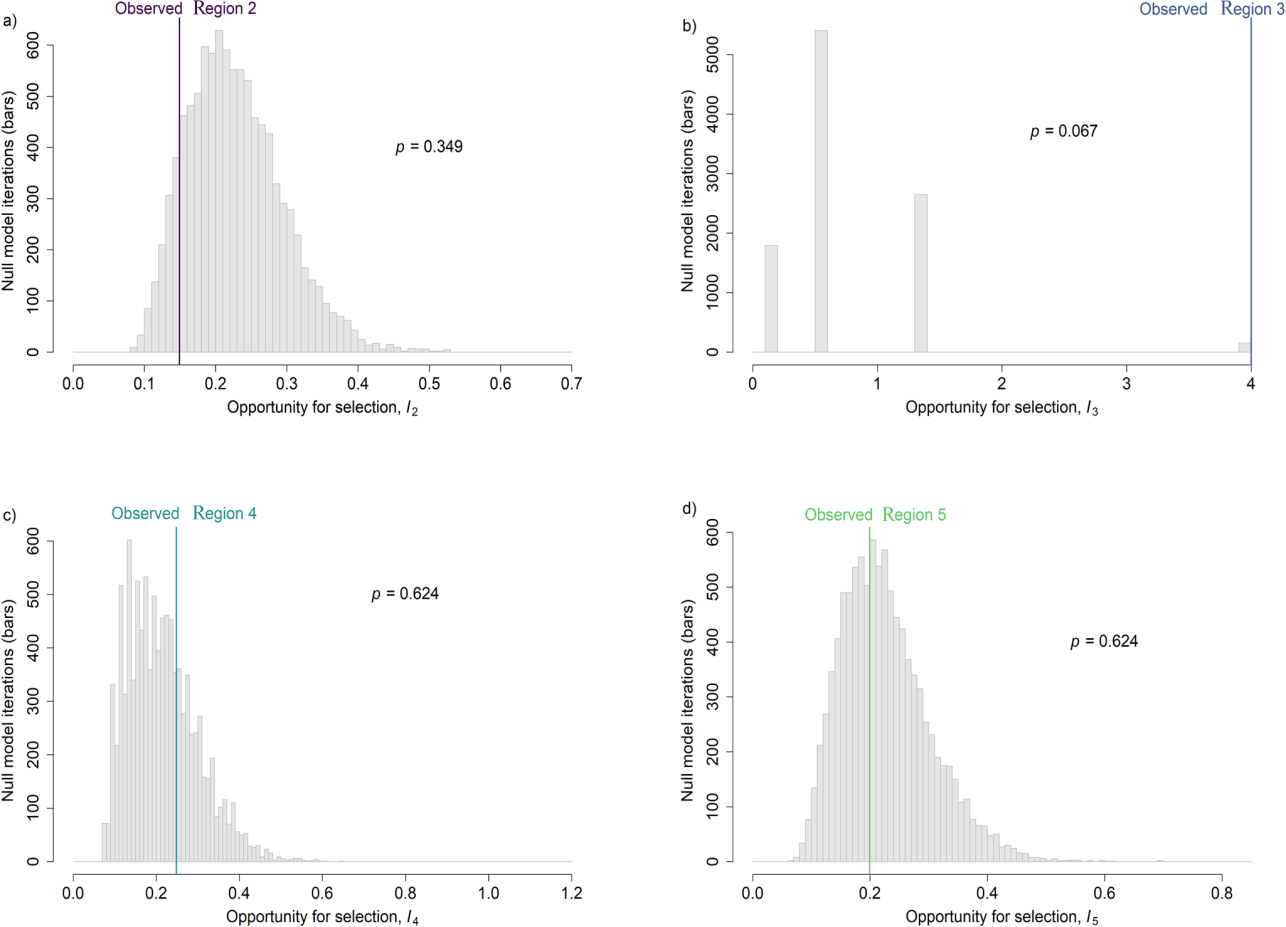
Observed opportunity for selection for maternal lines of *Quercus arkansana* saplings sourced from (a) Region 2, (b) Region 3, (c) Region 4, and (d) Region 5 after 10.5 months in an ex situ collection at the Oertli Family Hardy Plant Nursery of the Missouri Botanical Garden (vertical lines, observed opportunity for selection estimated using Equation 3; gray bars, null distributions according to the null model of demographic stochasticity; *p* values [adjusted], probability of a value at least as extreme as the observed value under the null model; region numbers follow Thomas et al. [2023]). Region 8 was omitted because only one maternal line was sourced from this region ($n_{r}$ = 1).

It was possible to calculate opportunity for selection, $I_{r}\left(t\right)$, for four out of the five regions represented in the ex situ collection of *Q*. *arkansana*. Only one maternal line was sourced from Region 8 (Figure 1); thus, the variance in relative fitness among maternal lines, $I_{r}\left(t\right)$ (Equation 3), could not be estimated. Among the four remaining regions, $I_{r}\left(t\right)$ ranged from 0.15 in Region 2 to 4 in Region 3 (Figure 4). The distribution of null $I_{r}\left(t\right)$ values differed markedly among regions, as expected from differences among regions in the number of maternal lines, $n_{r}$; initial individuals per maternal line, $N{\left(0\right)}_{ri}$; and observed mean survival rates across maternal lines $\bar{S_{r}}\left(t\right)$ (Appendix S1). Observed $I_{r}\left(t\right)$ for Regions 2, 3, 4, and 5 were not significantly different (adjusted *p* = 0.349, 0.067, 0.624, and 0.624, respectively) from the respective null distributions (Figure 4).

We found no statistically significant spatial autocorrelation in individual plant survival or in the residuals of the logistic regression of individual plant survival on maternal line (Appendix S2). Therefore, spatial environmental variation within the hoop house seemed not to have an effect on individual plant survival.

## DISCUSSION

As human pressures on biodiversity intensify, ex situ collections will likely be increasingly needed to temporarily protect plant species from threats experienced by wild populations (Smith & Pence, 2017) and provide plant material for conservation translocations (Westwood et al., 2021). In this context, the amount of intraspecific variation represented in an ex situ collection and how it declines over time due to plant death are central concerns (Guerrant & Fielder, 2004; Hoban, 2019; Thomas et al., 2023). We believe our approach is widely applicable for determining whether maternal line selection, due to differential survival, contributes to such decline. Our approach tests for provenance selection of maternal lines and opportunity for selection among maternal lines within provenances with null models based on demographic stochasticity.

Our approach focuses on maternal line selection, as measured by differential survival of siblings among maternal lines, because maternal lines constitute a practical and important partition of intraspecific variation in ex situ plant collections for conservation, as discussed above. Nonetheless, this focus means that the approach will not detect selection on traits that vary mainly within maternal lines rather than among maternal lines. For instance, the results for the empirical example with *Q*. *arkansana* showed no provenance selection or opportunity for selection beyond what would be expected by demographic stochasticity. However, these results would not necessarily inform practitioners about selection on paternally inherited traits, given that oaks reproduce sexually and are often self‐incompatible (Ducousso et al., 1993). This is a particular instance of a general problem common in studies of selection, namely, selection on unmeasured traits (Walsh & Lynch, 2018).

Another key feature of our approach is its emphasis on provenance. The approach focuses on detecting differential survival of maternal lines among provenance regions and opportunity for selection within such regions. This emphasis follows from the significance of provenance in efforts to represent intraspecific variation in ex situ collections (Griffith et al., 2021; Hoban, 2019; Hoban et al., 2020) and the fact that provenance data are often available in ex situ collections for conservation. Because local adaptation is common in plants (Baughman et al., 2019; Lascoux et al., 2016; Leimu & Fischer, 2008; Wadgymar et al., 2022), selection due to differential survival in ex situ collections may often operate on phenotypic features associated with provenance (Ignazi et al., 2020; König, 2005; Morgenstern, 1996; Risk et al., 2021; Wright, 1976), which in principle can be detected by analyses of differential survival of maternal lines among provenance regions. In addition, selection in ex situ collections may also operate on phenotypic features unrelated to provenance. In this case, selection can be detected by analyses of opportunity for selection within provenances, as proposed here, except when it operates on traits that vary mainly within (rather than among) maternal lines, as mentioned above. A crucial advantage of analyses of opportunity for selection is that no assumptions are required about the traits under selection (Walsh & Lynch, 2018).

Given the key role of provenance data in structuring our approach, the results produced and their interpretation depend on the definition of provenance regions (r in Equations (1), (2), (3)). As illustrated by the empirical example with *Q*. *arkansana*, such regions may be defined as rough estimates of distinct populations (Beckman et al., 2019), likely divergent in neutral or adaptive traits. An underlying assumption is that it is reasonable to treat provenance data as a categorical variable (i.e., geographic regions in Equations (1), (2), (3)), as is often done to describe variation across the geographic range of species for conservation purposes (e.g., (Beckman et al., 2019). Although we defined provenance regions for *Q. arkansana* based on an approximation to pollen dispersal distance (Thomas et al., 2023), provenance regions may also be delimited according to landscape barriers, climatic or genetic data, and, generally, information relevant to the structure of intraspecific variation across the geographic range of the focal species (e.g., Erickson & Halford, 2020). Variations in the methods proposed here could adopt continuous representations of provenance data. For example, it might be useful to examine how the fitness of maternal lines in an ex situ collection, measured by sibling survival, relates to the geographic coordinates (latitude, longitude, or both) of wild maternal plants. Such analyses could be regarded as estimates of fitness functions (Walsh & Lynch, 2018) in which the geographic coordinates of the maternal plants serve as surrogate for phenotypic traits that may determine survival in ex situ collections. Similarly, opportunity for selection could be measured across all maternal lines irrespective of provenance, bypassing the need to delineate provenance regions.

Together with provenance data, the period over which survival is measured influences the results produced by the methods proposed here and their interpretation. The relevant time span within which to measure survival (t in Equations (1), (2), (3)) depends on the kind of ex situ collection and concerns of their managers. Seed banks may require viability tests every few years (Center for Plant Conservation, 2019), whereas collections of whole growing plants may need more frequent assessments of survival, particularly over periods of potentially stressful conditions, such as cold or dry seasons. Estimates of selection, or opportunity for selection, for nonoverlapping periods may be combined to gauge the importance of different episodes of selection (Arnold & Wade, 1984; Walsh & Lynch, 2018).

The null models representing demographic stochasticity are key components of formal tests of selection and opportunity for selection (Gotelli & McGill, 2006; Walsh & Lynch, 2018; Waples & Reed, 2023). Without them, it would be difficult to assess the biological and statistical significance of observed values of provenance selection (Equation 1) and opportunity for selection (Equation 3). When applied to the empirical example with *Q*. *arkansana*, these null models produced a wide range of $\bar{S_{r}}\left(t\right)$ and $I_{r}\left(t\right)$ values, showing that substantial observed differences in survival (Figure 3) and opportunity for selection (Figure 4) could be explained by demographic stochasticity, as suggested by previous studies (Cabana & Kramer, 1991; Reed et al., 2023). Moreover, the patterns of variation produced by the null models showed that in general, the raw observed values of $\bar{S_{r}}\left(t\right)$ and $I_{r}\left(t\right)$ could not be directly compared across regions or ex situ collections because their respective null distributions depended on the mean survival across all provenance regions, the number of maternal lines, and the number of plantings per maternal line (Figures 3 & 4; Appendix S1). For example, under the null model of demographic stochasticity for maternal lines developed here, the expected magnitude of opportunity for selection and its sampling variance were negatively related to mean fitness (Appendix S1), consistent with the results of previous work (Reed et al., 2023).

Our method can be applied to ex situ collections that meet three criteria commonly regarded as key features in the context of plant conservation (Center for Plant Conservation, 2019): maternal lines sourced from multiple geographic regions, multiple maternal lines per geographic region, and multiple individual plants per maternal line. Even if not all three criteria are met, some of the methods may still be applicable, as illustrated by the empirical example with *Q*. *arkansana*. Analyses of selection can be conducted even in the absence of multiple maternal lines per region if the collection includes maternal lines from different regions (e.g., region 8, Figure 3). Additionally, opportunity for selection can be examined in ex situ collections holding multiple maternal lines from a single geographic region, as long as at least some maternal lines are represented by multiple individual plants.

An important assumption of our approach is that the ex situ collection of the focal species is a de facto common garden experiment (Wadgymar et al., 2022). Deviations from this assumption can result in differential survival among maternal lines that mimic provenance selection but, in reality, reflect differences in ex situ environmental conditions among plants from different provenances. Similarly, a seemingly large opportunity for selection, beyond what is expected from demographic stochasticity, can be an artifact of variation in ex situ environmental conditions among maternal lines from a single provenance. These are instances of environmentally generated correlations between fitness and traits, a well‐known problem in studies of selection (Walsh & Lynch, 2018). As shown in our example with *Q*. *arkansana*, the effect of spatial heterogeneity in ex situ environmental conditions can be statistically controlled through randomization of the location of individual plants in the ex situ collection, independent of maternal line and provenance. Moreover, the assumption that individual survival is unrelated to spatial heterogeneity under ex situ environmental conditions can be examined by testing for spatial autocorrelation in individual survival (Appendix S2).

Provenance and survival data are indispensable for implementation of our method. Provenance data are common in ex situ collections but often incomplete (Thomas et al., 2022, 2023). Survival data may be less common, but collecting it would ideally be standard practice in ex situ collections of threatened species, given its importance for collection management (Chau et al., 2019; de Andrade et al., 2021; Godefroid et al., 2010; Thomas et al., 2023). These data are crucial to the understanding of survival patterns in ex situ collections, including maternal line selection and the effects of spatial and temporal environmental variation. Enhancing the quality of plant records through the incorporation of provenance details and timely documentation of mortality and the cause of death represents an important step managers of ex situ collections can take that can enable novel analytical approaches that support conservation efforts (Thomas et al., 2024).

Selection due to differential survival is widely acknowledged as a process likely to reduce intraspecific variation in ex situ plant collections, which diminishes the value of these collections as safeguards against extinction of wild populations and as sources of plant material for conservation translocations (Ensslin & Godefroid, 2019; Havens et al., 2004; Rauschkolb et al., 2019). However, despite a few notable studies (Galíndez et al., 2019; Nagel et al., 2015), formal assessments of selection in ex situ plant collections seem scarce. We hope our method will help botanical garden staff, seed bank curators, and conservation practitioners make decisions about ex situ conservation programs, including how collections are maintained, which maternal lines are preserved, and what strategies are implemented to maintain intraspecific diversity within their collections. These professionals can use provenance and survival data, as we have proposed, to better understand the extent to which selection contributes to the decline in intraspecific variation through time. Given such decline is commonly observed in these collections (Wei & Jiang, 2021), our method can inform management strategies aimed at maintaining a broad representation of the intraspecific variation in imperiled species.

## Supporting information



Supporting Information

Supporting Information
