# Supplementary material for: Measuring maternal line selection driven by differential survival in ex situ collections for plant conservation
Source: Conserv Biol. 2026 Jul 3;40(4):e70350. doi: 10.1111/cobi.70350 (PMC13392728; doi:10.1111/cobi.70350)

**Appendix S1: Properties of the distribution of** $\bar{S_{r}}\left( t \right)$ **and** $I_{r}\left( t \right)$

**under null models based on demographic stochasticity**

In this appendix, we first described the mean and variance of the distribution of $\bar{S_{r}}\left( t \right)$ under the null model for provenance selection that, as described in the main text, was based on demographic stochasticity. $\bar{S_{r}}\left( t \right)$ is the mean survival rate of individual plants across maternal lines sourced from region $r$, as described in the main text (equation 1). Then, in the second section of this appendix, we described the mean and variance of the distribution $I_{r}\left( t \right)$ under the null model for opportunity for selection. This null model was also based on demographic stochasticity. $I_{r}\left( t \right)$ is the variance in relative fitness among maternal lines sourced from region $r$, as described in the main text (equation 3). Except for terms defined in this appendix, all notations followed Table 1. The references cited in the text below are listed at the end, before the five figures (Figures S1 ̶ S5). The code for all simulations described in this appendix is available at <https://github.com/mobot-living-collections/ex-situ-selection>.

**1) Mean and variance of the distribution of** $\bar{S_{r}}\left( t \right)$ **under the null model of demographic stochasticity for provenance selection.**

As explained in the main text, the null model for provenance selection randomly distributes individual plant deaths among maternal lines $(i$), irrespective of provenance region ($r)$. It preserves the initial number of individual plants and the number of individual plant deaths observed across the whole ex situ collection over time $t$. Therefore, the null model also preserves the total number of plants surviving in the ex situ collection. Here after we use $K$ to refer to this total number of plants surviving after time span $t$ and $N$ to refer to the total number of initial plants in the ex situ collection:

$K=\sum_{r=1}^{m} \sum_{i=1}^{n_{r}} N_{ri}\left( t \right)$ (S1),

$N=\sum_{r=1}^{m} \sum_{i=1}^{n_{r}} N_{ri}\left( 0 \right)$ (S2),

where $m$ is the total number of regions represented in the ex situ collection and all other terms on the right-hand side of the equations S1 and S2 are as defined in Table 1.

At each iteration of the null model, the value of $N_{ri}\left( t \right)$ corresponds to a count of surviving plants among $N_{ri}\left( 0 \right)$random draws from a finite population of $N$ plants that only include $K$ surviving plants. Thus, the distribution of the null values of $N_{ri}\left( t \right)$ can be described by the hypergeometric distribution (Forbes et al. 2011), $N_{ri}\left( t \right) \sim Hypergeometric\left( N,K, N_{ri}\left( 0 \right) \right)$, with mean $N_{ri}\left( 0 \right)\frac{K}{N}$ and variance $N_{ri}\left( 0 \right) \frac{K}{N}\frac{N-K}{N}\frac{N-N_{ri}\left( 0 \right)}{N-1}$. It follows that the expected value of the null values of $S_{ri}\left( t \right)$, across iterations of the null model, is:

E$\left[ S_{ri}\left( t \right) \right]=\frac{K}{N}$ (S3).

Likewise, the variance of the null values of $S_{ri}\left( t \right),$ across iterations of the null model, is:

Var$\left[ S_{ri}\left( t \right) \right]=\frac{1}{N_{ri}\left( 0 \right)} \frac{K}{N}\frac{N-K}{N}\frac{N-N_{ri}\left( 0 \right)}{N-1}$ (S4).

In turn, equations S3 and S4 imply that the expected null value of $\bar{S_{r}}\left( t \right)$, across iterations of the null model, is:

E$\left[ \bar{S_{r}}\left( t \right) \right]=\frac{K}{N}$ (S5).

Equation S5 shows that the expected value of $\bar{S_{r}}\left( t \right)$, E$\left[ \bar{S_{r}}\left( t \right) \right]$, does not vary with $N_{ri}\left( 0 \right)$ or $n_{r}$.

On the other hand, the variance of the null value of $\bar{S_{r}}\left( t \right)$ is a function of the sum of variances of all $S_{ri}\left( t \right)$ and the respective covariances:

Var$\left[ \bar{S_{r}}\left( t \right) \right]=\frac{1}{n_{r}^{2}}\sum_{i=1}^{nr} Var\left[ S_{ri}\left( t \right) \right]+\frac{2}{n_{r}^{2}}\sum_{1\leq i<j\leq n_{r}} Cov\left[ S_{ri}\left( t \right), S_{rj}\left( t \right) \right]$ (S6).

There are $\left( \begin{matrix} n_{r} \\ 2 \end{matrix} \right)$ covariances terms in S6, which equals a total of $\frac{n_{r}\left( n_{r}-1 \right)}{2}$ covariance terms. Each one of these covariance terms is:

$Cov\left[ S_{ri}\left( t \right), S_{rj}\left( t \right) \right]=\frac{1}{N_{ri}\left( 0 \right)N_{rj}\left( 0 \right)}Cov[N_{ri}\left( t \right), N_{rj}\left( t \right)]$,

where

$Cov\left[ N_{ri}\left( t \right), N_{rj}\left( t \right) \right]=-N_{ri}\left( 0 \right)N_{rj}\left( 0 \right)\frac{K}{N}\frac{N-K}{N}\frac{1}{N-1}$.

Thus, S6 may be expressed as:

Var$\left[ \bar{S_{r}}\left( t \right) \right]=\frac{K}{N}\frac{N-K}{N}\frac{1}{\left( N-1 \right)n_{r}^{2}}\left( \sum_{i=1}^{n_{r}} \frac{N-N_{ri}\left( 0 \right)}{N_{ri}\left( 0 \right)}- n_{r}\left( n_{r}-1 \right) \right)$ (S7).

Equation S7 shows that Var$\left[ \bar{S_{r}}\left( t \right) \right]$ is high for intermediate survival probabilities ($K/N$=0.5) and decreases towards lower and higher survival probabilities. It also shows that Var$\left[ \bar{S_{r}}\left( t \right) \right]$decreases with increasing $N_{ri}\left( 0 \right)$ and, also, with increasing $n_{r}$. Note also that variation among maternal lines in $N_{ri}\left( 0 \right)$ values likely affects Var$\left[ \bar{S_{r}}\left( t \right) \right]$, given that equation S7 does not subsumes $N_{ri}\left( 0 \right)$ values for different maternal lines into a single value across the region, such as $\sum_{i=1}^{n_{r}} N_{ri}\left( 0 \right)$.

Next, we used two simulations to explore the properties of E$\left[ \bar{S_{r}}\left( t \right) \right]$ and Var$\left[ \bar{S_{r}}\left( t \right) \right]$ implied in equations S5 and S7, respectively. The first simulation aimed to explore the effects of survival probability ($K/N$), initial number of plants per maternal line ($N_{ri}\left( 0 \right)$) and number of maternal lines ($n_{r}$) on E$\left[ \bar{S_{r}}\left( t \right) \right]$ and Var$\left[ \bar{S_{r}}\left( t \right) \right]$. In particular, we simulated null values of $\bar{S_{r}}\left( t \right)$ under 54 combinations of the levels of three variables: two values of $n_{r}$ (10, 20), three values of $N_{ri}\left( 0 \right)$ (2, 10, 20) and nine probabilities of survival, $K/N$ (from 0.1 to 0.9 every 0.1), with total number of plants in the ex situ collection fixed at $N=$ 800. For each of the 54 combinations of the levels of three variables, we ran 10,000 iterations of the null model based on demographic stochasticity to calculate E$\left[ \bar{S_{r}}\left( t \right) \right]$ and Var$\left[ \bar{S_{r}}\left( t \right) \right]$. Additionally, for each of the 54 combinations of the levels of three variables, we estimated the length of the 95% confidence interval for the null distribution of $\bar{S_{r}}\left( t \right)$. To estimate the length of this interval we calculated the difference between the 97.5 and 2.25 percentiles of the null distribution of $\bar{S_{r}}\left( t \right)$.

The second simulation aimed to explore the effect that variation among maternal lines in $N_{ri}\left( 0 \right)$ values may have on Var$\left[ \bar{S_{r}}\left( t \right) \right]$, suggested by the fact that, in equation S7, $N_{ri}\left( 0 \right)$ values are not subsumed into a single value across each region. We simulated null values of $\bar{S_{r}}\left( t \right)$ under 108 combinations of the levels of four variables: three values of $N_{r}$ (100, 200, 400), two values of $n_{r}$ (10, 50), two levels of evenness in the distribution of plants among maternal lines (even and uneven) and nine probabilities of survival, $K/N$ (from 0.1 to 0.9 every 0.1) with total number of plants in the ex situ collection fixed at $N=$ 800. When the distribution of plants among maternal lines was even, all maternal lines had $N_{ri}\left( 0 \right)=\frac{N_{r}}{n_{r}}$. In contrast, when the distribution of plants among maternal lines was uneven, half of the maternal lines had $N_{ri}\left( 0 \right)=\frac{1.5\times N_{r}}{n_{r}}$ and the other half $N_{ri}\left( 0 \right)=\frac{0.5\times N_{r}}{n_{r}}$. For each of the 108 combinations of the levels of four variables, we ran 10,000 iterations of the null model based on demographic stochasticity to calculate E$\left[ \bar{S_{r}}\left( t \right) \right]$ and Var$\left[ \bar{S_{r}}\left( t \right) \right]$. For each of the 108 combinations of the levels of four variables, we also estimated the length of the 95% confidence interval for the null distribution of $\bar{S_{r}}\left( t \right)$, as described above.

The results of both simulations confirm that E$\left[ \bar{S_{r}}\left( t \right) \right]=\frac{K}{N}$ regardless of $N_{ri}\left( 0 \right)$ or $n_{r}$ values (Fig. S1 a, c, e and Fig. S3 a, c, e). They also showed that Var$\left[ \bar{S_{r}}\left( t \right) \right]$ was high for intermediate survival probabilities ($K/N$=0.5) and decreased towards lower and higher survival probabilities (Fig. S1 b, d, f and Fig. S3 b, d, f). Also, Var$\left[ \bar{S_{r}}\left( t \right) \right]$ decreased as a result of increasing $N_{ri}\left( 0 \right)$ or $n_{r}$ values (Fig. S1 b, d, f). Likewise, Var$\left[ \bar{S_{r}}\left( t \right) \right]$ decreased with increasing $N_{r}$ values (Fig. S3 b, d, f). Finally, Var$\left[ \bar{S_{r}}\left( t \right) \right]$ decreased as a result of decreasing variation among maternal lines in $N_{ri}\left( 0 \right)$ values while keeping $N_{r}$ values fixed (Fig. S3 b, d, f). Depending on the combination of parameter values, the length of the 95% confidence interval for the null distribution of $\bar{S_{r}}\left( t \right)$ can be a substantial portion of the full range of values that $\bar{S_{r}}\left( t \right)$ may take (Figs. S2 and S4).

**2) Mean and variance of** $I_{r}\left( t \right)$ **under the null model of demographic stochasticity for opportunity for selection.**

As described in the main text, the null model for opportunity for selection within regions, $I_{r}\left( t \right)$, randomly distributes individual plant deaths among maternal lines $(i$) within a provenance region ($r)$. It preserves the number of individual plant deaths observed over time $t$ across the whole ex situ collection, and in each provenance region ($r)$. Therefore, the null model also preserves the total number of plants sourced from region ($r)$that survived. Here after we use $K_{r}$ to refer to the total number of plants sourced from region ($r)$ that survived after time span $t$ and $N_{r}$ to refer to the total number of initial plants sourced from region ($r)$:

$K_{r}=\sum_{i=1}^{n_{r}} N_{ri}\left( t \right)$ (S8),

$N_{r}=\sum_{i=1}^{n_{r}} N_{ri}\left( 0 \right)$ (S9).

At each iteration of the null model, the value of $N_{ri}\left( t \right)$ corresponds to a count of surviving plants among $N_{ri}\left( 0 \right)$random draws from a finite population of $N_{r}$ plants that only include $K_{r}$ surviving plants. Thus, the distribution of the null values of $N_{ri}\left( t \right)$ can be described by the hypergeometric distribution $N_{ri}\left( t \right) \sim Hypergeometric\left( N_{r} ,K_{r}, N_{ri}\left( 0 \right) \right)$, with mean $N_{ri}\left( 0 \right) \frac{K_{r}}{N_{r}}$ and variance $N_{ri}\left( 0 \right) \frac{K_{r}}{N_{r}}\frac{N_{r}-K_{r}}{N_{r}}\frac{N_{r}-N_{ri}\left( 0 \right)}{N_{r}-1}$.

Approaches to obtain closed expressions for the mean and variance of the null distribution of $I_{r}\left( t \right)$ seem to require assumptions that are unlikely to be met in many practical cases. By example, the delta method (Casella and Berger 2002) works reasonably well under the assumption that the variance among null values of $\bar{S_{r}}\left( t \right)$, generated across iterations of the null model, is low. Yet, high variation in $n_{r}$ and small values of $N_{ri}\left( 0 \right)$ may be common in living collections and cause high variance among null values of $\bar{S_{r}}\left( t \right)$. Therefore, rather than relying on methods to approximate the mean and variance of the null distribution of $I_{r}\left( t \right)$, we used simulations to explore properties of this distribution.

We simulated null values of $I_{r}\left( t \right)$ under 108 combinations of the levels of four variables: three values of $N_{r}$ (100, 200, 400), nine probabilities of survival, $\frac{K_{r}}{N_{r}}$ (from 0.1 to 0.9 every 0.1), two values of $n_{r}$ (10, 50), and two levels of evenness in the distribution of plants among maternal lines (even and uneven). When the distribution of plants among maternal lines was even, all maternal lines had $N_{ri}\left( 0 \right)=\frac{N_{r}}{n_{r}}$. In contrast, when the distribution of plants among maternal lines was uneven, half of the maternal lines had $N_{ri}\left( 0 \right)=\frac{1.5\times N_{r}}{n_{r}}$ and the other half $N_{ri}\left( 0 \right)=\frac{0.5\times N_{r}}{n_{r}}$. For each of the 108 combinations of the levels of four variables, we ran 10,000 iterations of the null model based on demographic stochasticity to calculate the mean (or expected value) and variance of $I_{r}\left( t \right)$, E$\left[ I_{r}\left( t \right) \right]$ and Var$\left[ I_{r}\left( t \right) \right]$, respectively.

The results of the simulations show that E$\left[ I_{r}\left( t \right) \right]$ decreased with increasing values of $N_{r}$ and survival probability, $\frac{K_{r}}{N_{r}}$ (Fig. S5 a, c, e). For a fixed $N_{r}$ value, E$\left[ I_{r}\left( t \right) \right]$ also decreased with decreasing number of maternal lines, $n_{r}$ (Fig. S5 a, c, e), because this reduction in the number of maternal lines increased the initial number of plants per maternal line, $N_{ri}\left( 0 \right).$ Finally, E$\left[ I_{r}\left( t \right) \right]$ decreased as the evenness of the distribution of plants among maternal lines increased (Fig. S5 a, c, e).

The results for Var$\left[ I_{r}\left( t \right) \right]$ showed similar trends to those described above for E$\left[ I_{r}\left( t \right) \right]$, but there were important differences. Var$\left[ I_{r}\left( t \right) \right]$ decreased with increasing values of $N_{r}$ and survival probability, $\frac{K_{r}}{N_{r}}$ (Fig. S5 b, d, f). For a fixed $N_{r}$ value, Var$\left[ I_{r}\left( t \right) \right]$ also decreased with decreasing number of maternal lines, $n_{r}$ (Fig. S5 b, d, f), again, because a lower number of maternal lines meant that the initial number of plants per maternal line, $N_{ri}\left( 0 \right)$, increased. Last, the evenness of the distribution of plants among maternal lines had complex effects on Var$\left[ I_{r}\left( t \right) \right]$. For the two highest values of $N_{r}$ we considered (200 and 400), Var$\left[ I_{r}\left( t \right) \right]$ decreased as the evenness of the distribution of plants among maternal lines increased (Fig. S5 d, f). This was also the case when $N_{r}=100$ and the number of maternal lines was low, $n_{r}=10$ (Fig. S5 b). However, when $N_{r}=100$ and $n_{r}=50$, Var$\left[ I_{r}\left( t \right) \right]$ did not consistently decreased as the evenness of the distribution of plants among maternal lines increased (Fig. S5 b). In fact, often it increased as the evenness of the distribution of plants among maternal lines increased.

Further study is needed to fully understand the relationship between the evenness of the distribution of plants among maternal lines and Var$\left[ I_{r}\left( t \right) \right]$. However, we suspect some of the complexity in this relationship may be explained by the number of plants per maternal line, $N_{ri}\left( 0 \right)$. When $N_{r}=100$ and $n_{r}=50$, $N_{ri}\left( 0 \right)$ may take values of 1, 2 or 3. Depending on the evenness of the distribution of plants among maternal lines, the distribution of null values of $\bar{S_{r}}\left( t \right)$ can have different shapes and variance despite a constant expected value (Fig. S3). Because $\bar{S_{r}}\left( t \right)$ is used to calculate $I_{r}\left( t \right)$, as shown by equation 3 in the main text, the shape of the distribution of $\bar{S_{r}}\left( t \right)$ affects the shape of the null distribution of $I_{r}\left( t \right)$ and thus its variance. This illustrates properties of the null distribution of $I_{r}\left( t \right)$ under parameter values that may be common in many applied instances and, yet, not captured by approximations (e.g., the delta method) that assume little variation among null values of $\bar{S_{r}}\left( t \right)$.

**References**

Casella, G. and Berger, R., 2002. Statistical inference. Second edition. Brooks/Cole, Cengage Learning.

Forbes, C., Evans, M., Hastings, N. and Peacock, B., 2011. Statistical distributions. John Wiley & Sons.

**Figure S1.** Mean (a, c, d) and variance (b, d, f) of the null distribution of $\bar{S_{r}}\left( t \right)$ for 54 combinations of the levels of three variables: two values of $n_{r}$ (10, 20), three values of $N_{ri}\left( 0 \right)$ (2, 10, 20) and nine probabilities of survival, $\frac{K}{N}$ (from 0.1 to 0.9 every 0.1), with total number of plants in the ex situ collection fixed at $N=$ 800. The mean of the null distribution $\bar{S_{r}}\left( t \right)$, E$\left[ \bar{S_{r}}\left( t \right) \right]$, is shown in the vertical axes of panels a, c and e. The variance of the null distribution $\bar{S_{r}}\left( t \right)$, Var$\left[ \bar{S_{r}}\left( t \right) \right]$, is shown in the vertical axes of panels b, d and f. Each value of E$\left[ \bar{S_{r}}\left( t \right) \right]$ and Var$\left[ \bar{S_{r}}\left( t \right) \right]$ was based on 10,000 iterations of the null model.


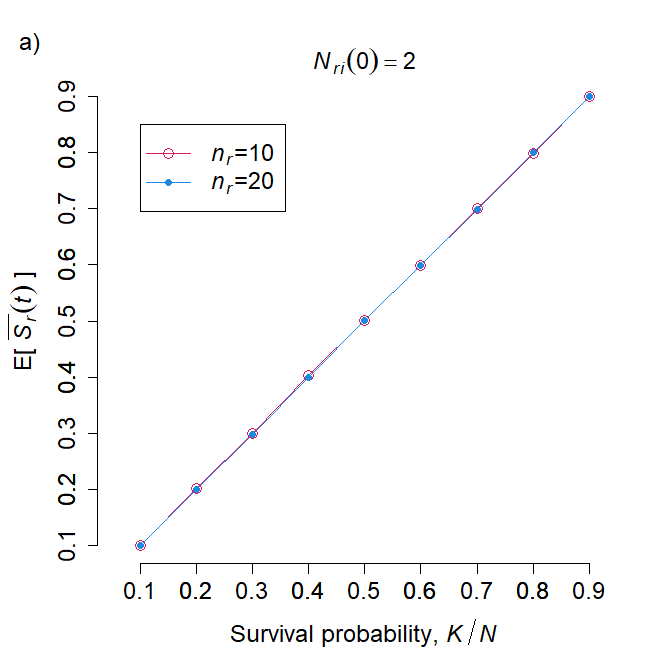

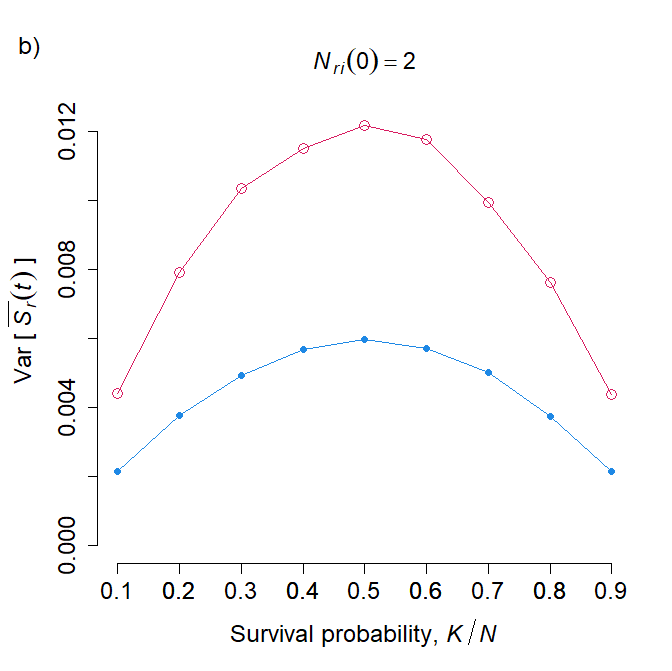


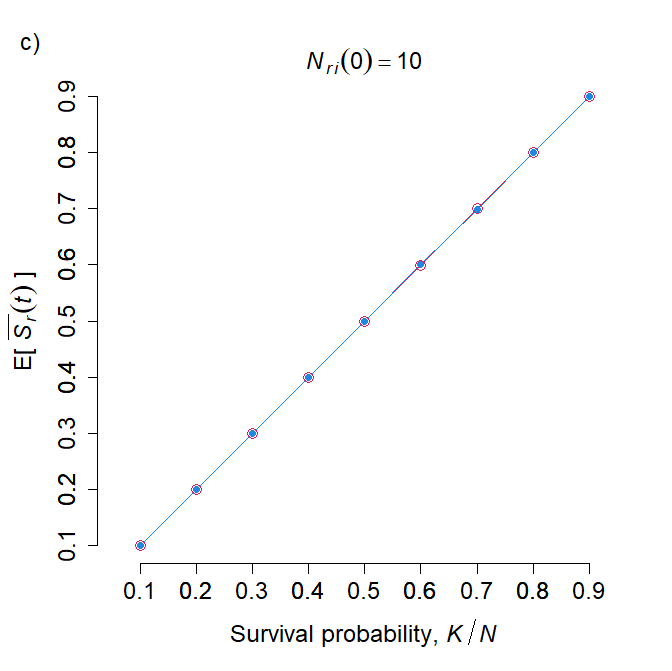

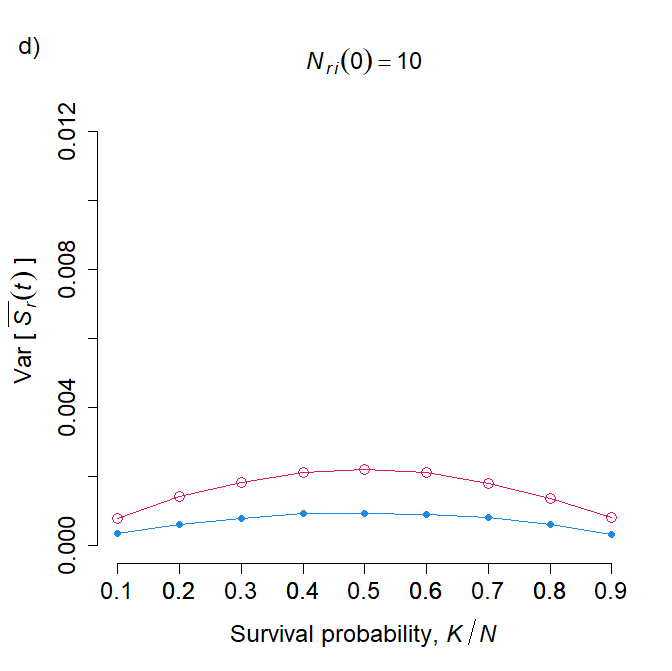


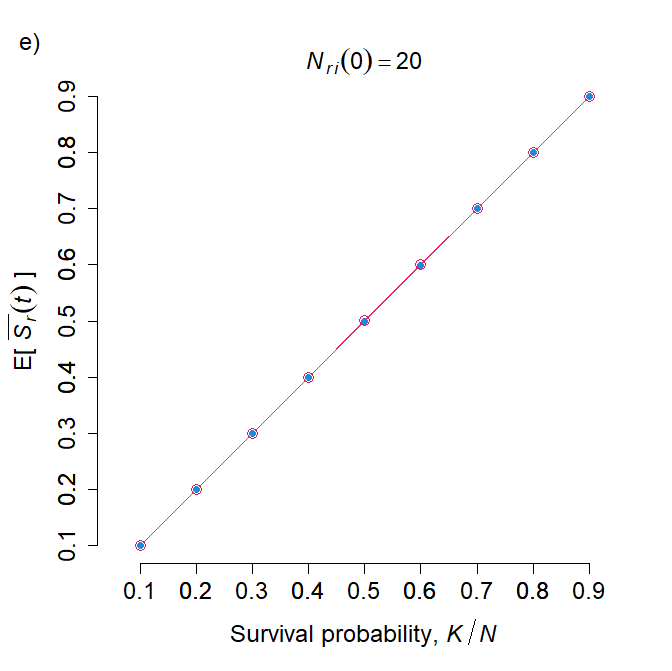

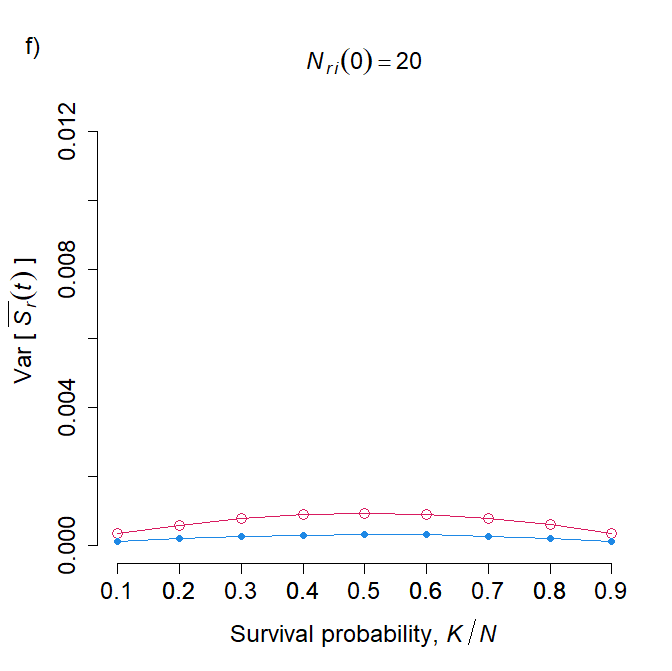


**Figure S2.** Length of the 95% confidence intervals of the null distribution of $\bar{S_{r}}\left( t \right)$ for 54 combinations of the levels of three variables: two values of $n_{r}$ (10, 20), three values of $N_{ri}\left( 0 \right)$ (2, 10, 20) and nine probabilities of survival, $\frac{K}{N}$ (from 0.1 to 0.9 every 0.1), with total number of plants in the ex situ collection fixed at $N=$ 800. Each point (showing the length of the 95% confidence intervals of the null distribution of $\bar{S_{r}}\left( t \right)$) was based on 10,000 iterations of the null model.


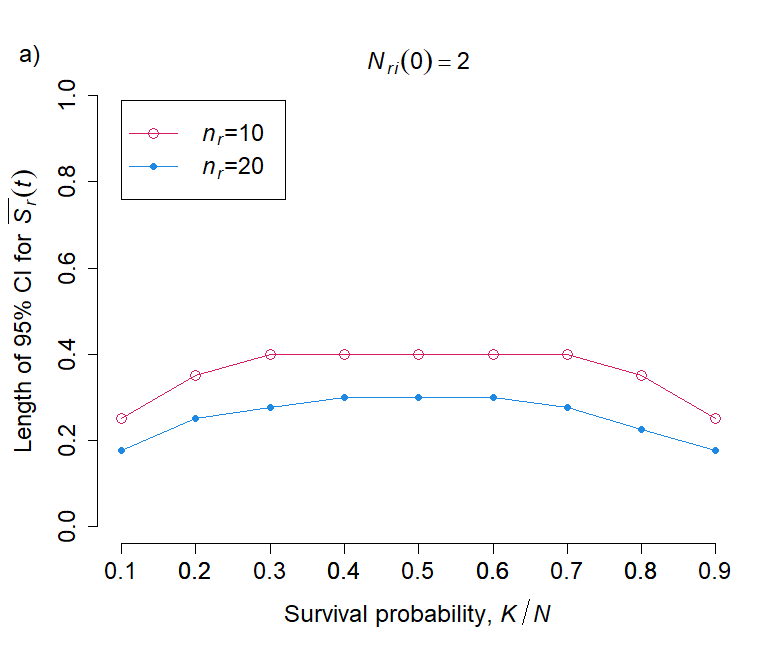


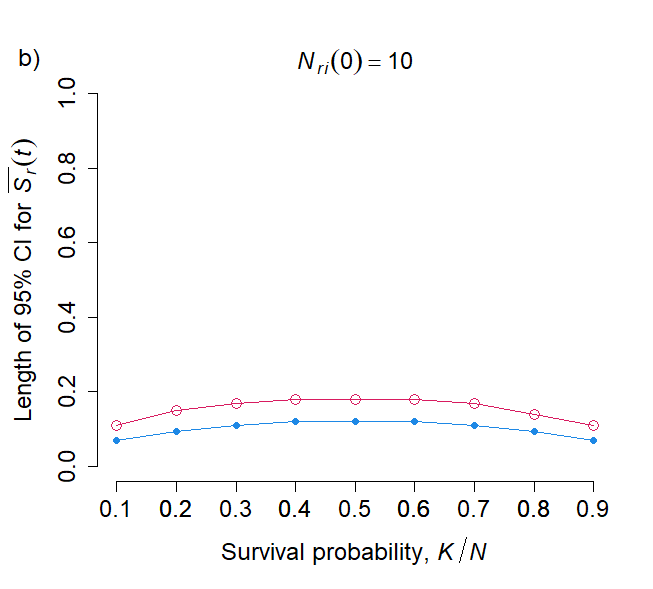


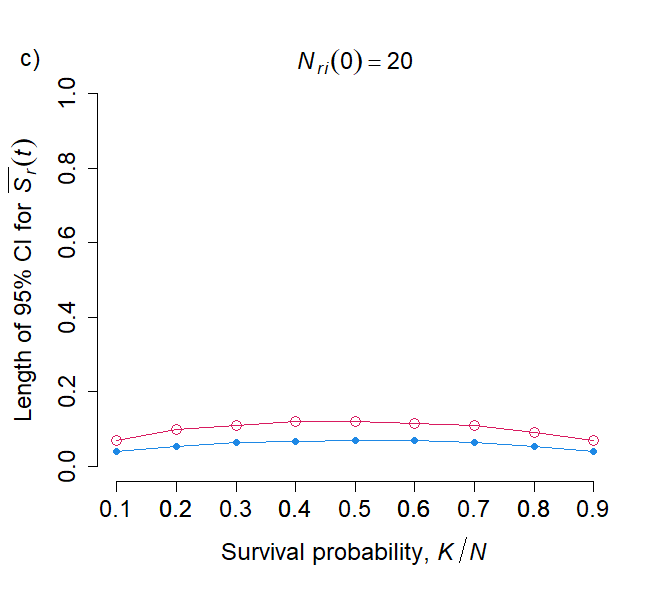


**Figure S3.** Mean (a, c, d) and variance (b, d, f) of the null distribution of $\bar{S_{r}}\left( t \right)$ for 108 combinations of the levels of four variables: three values of $N_{r}$ (100, 200, 400), two values of $n_{r}$ (10, 50), two levels of evenness in the distribution of plants among maternal lines (even and uneven) and nine probabilities of survival, $\frac{K}{N}$ (from 0.1 to 0.9 every 0.1) with total number of plants in the ex situ collection fixed at $N=$ 800. When the distribution of plants among maternal lines was even, all maternal lines had $N_{ri}\left( 0 \right)=\frac{N_{r}}{n_{r}}$. In contrast, when the distribution of plants among maternal lines was uneven, half of the maternal lines had $N_{ri}\left( 0 \right)=\frac{1.5\times N_{r}}{n_{r}}$ and the other half $N_{ri}\left( 0 \right)=\frac{0.5\times N_{r}}{n_{r}}$. The mean of the null distribution $\bar{S_{r}}\left( t \right)$, E$\left[ \bar{S_{r}}\left( t \right) \right]$, is shown in the vertical axes of panels a, c and e. The variance of the null distribution $\bar{S_{r}}\left( t \right)$, Var$\left[ \bar{S_{r}}\left( t \right) \right]$, is shown in the vertical axes of panels b, d and f. Each value of E$\left[ \bar{S_{r}}\left( t \right) \right]$ and Var$\left[ \bar{S_{r}}\left( t \right) \right]$ was based on 10,000 iterations of the null model.


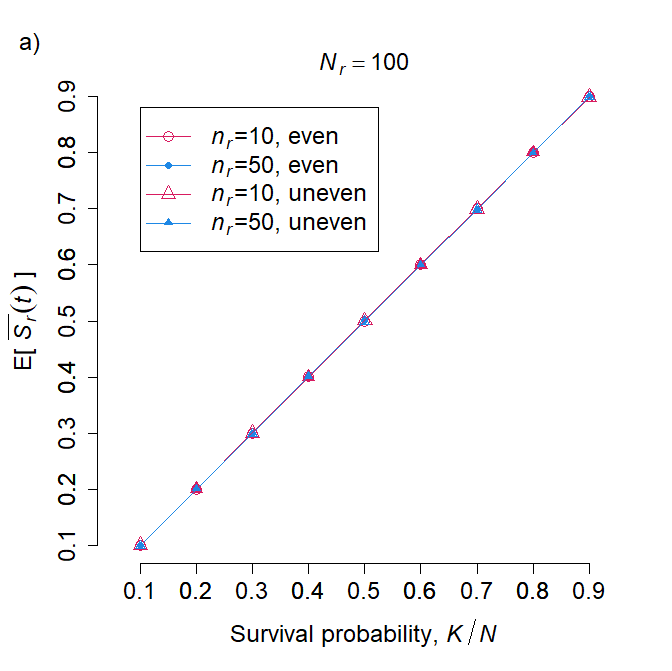

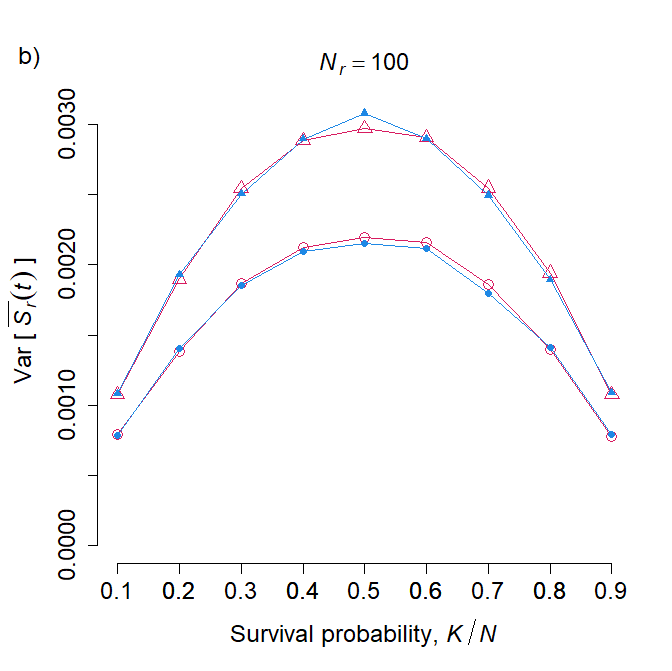


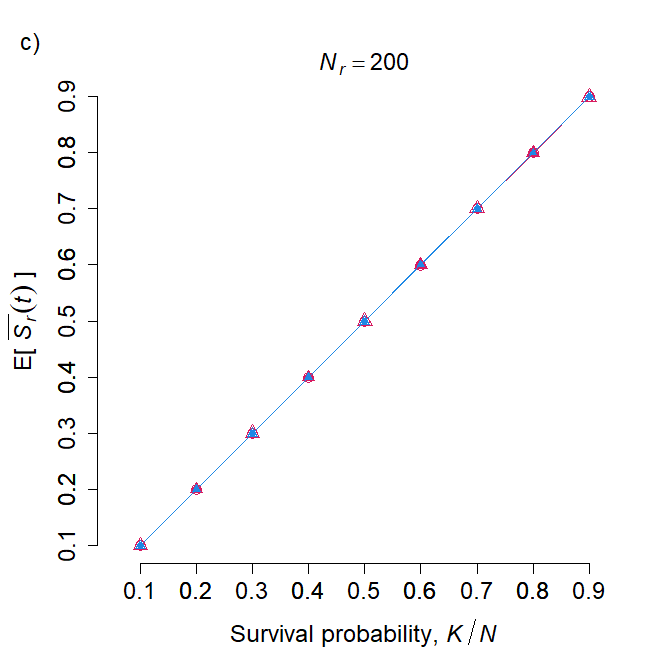

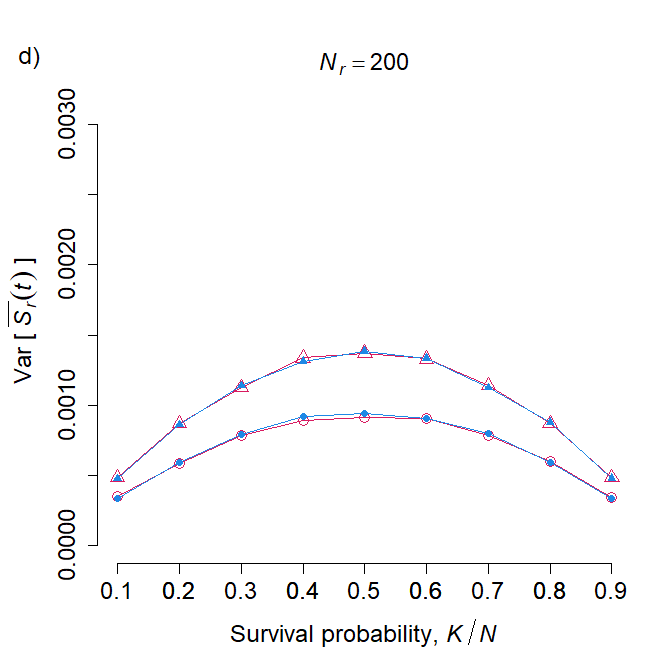


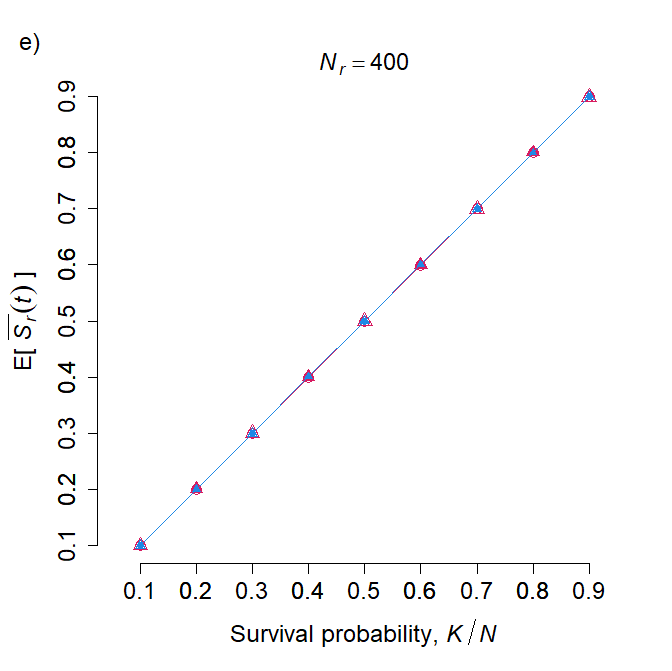
**
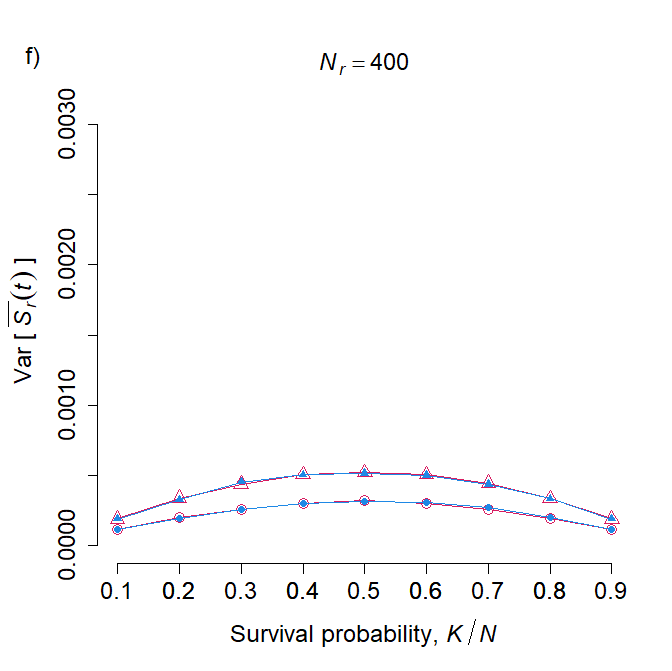
**

**Figure S4.** Length of the 95% confidence intervals of the null distribution of $\bar{S_{r}}\left( t \right)$ for 108 combinations of the levels of four variables: three values of $N_{r}$ (100, 200, 400), two values of $n_{r}$ (10, 50), two levels of evenness in the distribution of plants among maternal lines (even and uneven) and nine probabilities of survival, $\frac{K}{N}$ (from 0.1 to 0.9 every 0.1) with total number of plants in the ex situ collection fixed at $N=$ 800. When the distribution of plants among maternal lines was even, all maternal lines had $N_{ri}\left( 0 \right)=\frac{N_{r}}{n_{r}}$. In contrast, when the distribution of plants among maternal lines was uneven, half of the maternal lines had $N_{ri}\left( 0 \right)=\frac{1.5\times N_{r}}{n_{r}}$ and the other half $N_{ri}\left( 0 \right)=\frac{0.5\times N_{r}}{n_{r}}$. Each point (showing the length of the 95% confidence intervals of the null distribution of $\bar{S_{r}}\left( t \right)$) was based on 10,000 iterations of the null model.


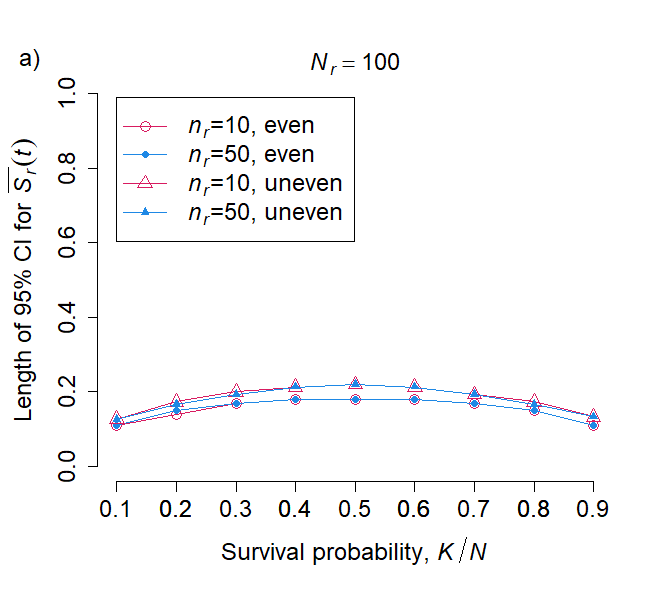


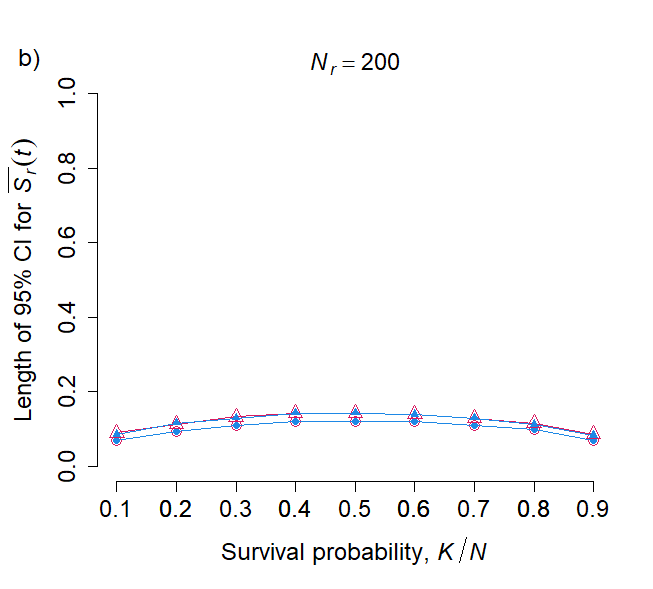


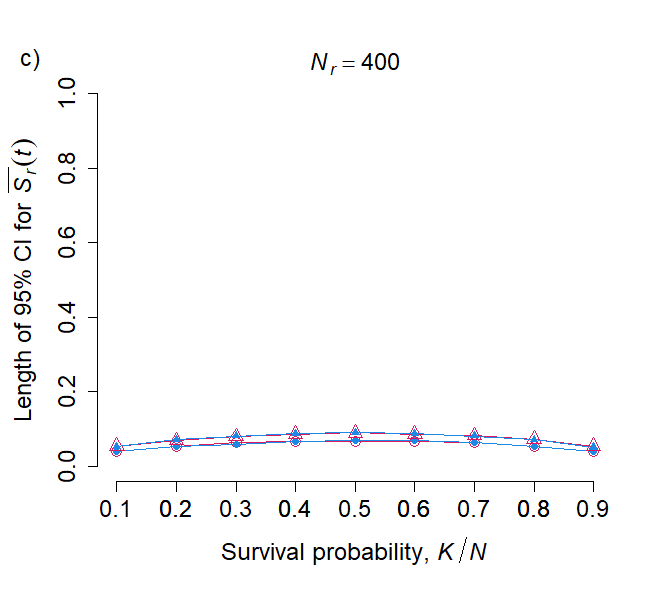


**Figure S5.** Mean (a, c, d) and variance (b, d, f) of the null distribution of $I_{r}\left( t \right)$ for 108 combinations of the levels of four variables: the total number of plants from a given region that are represented in an ex situ collection, $N_{r}$, probabilities of survival, $\frac{K_{r}}{N_{r}}$ (in the horizontal axis of each panel), the number of maternal lines, $n_{r}$, and two levels of evenness in the distribution of plants among maternal lines. When the distribution of plants among maternal lines was even, all maternal lines had an initial number of plants $N_{ri}\left( 0 \right)=\frac{N_{r}}{n_{r}}$. In contrast, when the distribution of plants among maternal lines was uneven, half of the maternal lines had $N_{ri}\left( 0 \right)=\frac{1.5\times N_{r}}{n_{r}}$ and the other half $N_{ri}\left( 0 \right)=\frac{0.5\times N_{r}}{n_{r}}$. The mean of the null distribution $I_{r}\left( t \right)$, E$\left[ I_{r}\left( t \right) \right]$, is shown in the vertical axes of panels a, c and e. The variance of the null distribution $I_{r}\left( t \right)$, Var$\left[ I_{r}\left( t \right) \right]$, is shown in the vertical axes of panels b, d and f. Each value of E$\left[ I_{r}\left( t \right) \right]$ and Var$\left[ I_{r}\left( t \right) \right]$ was based on 10,000 iterations of the null model.


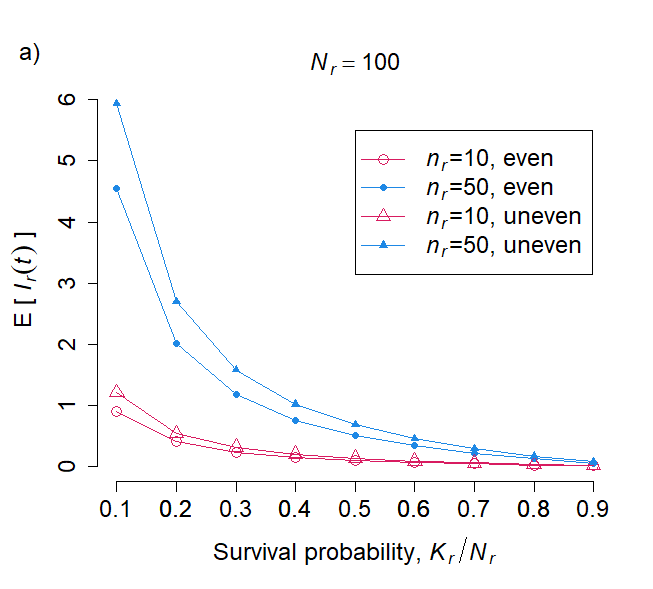

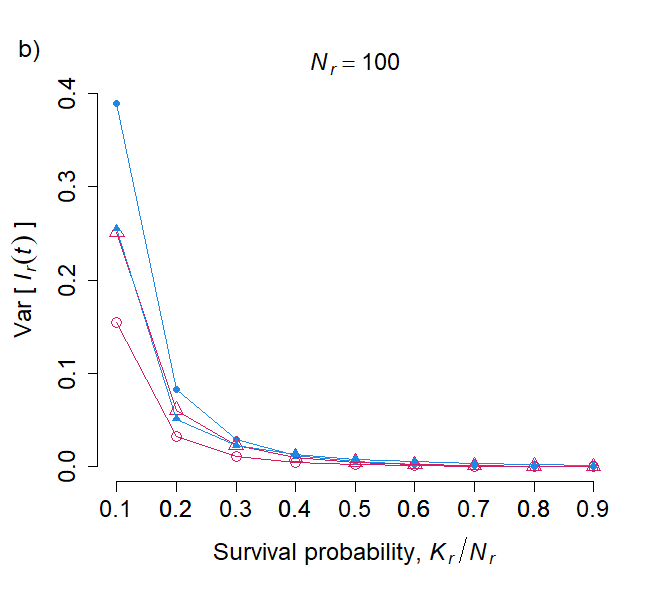


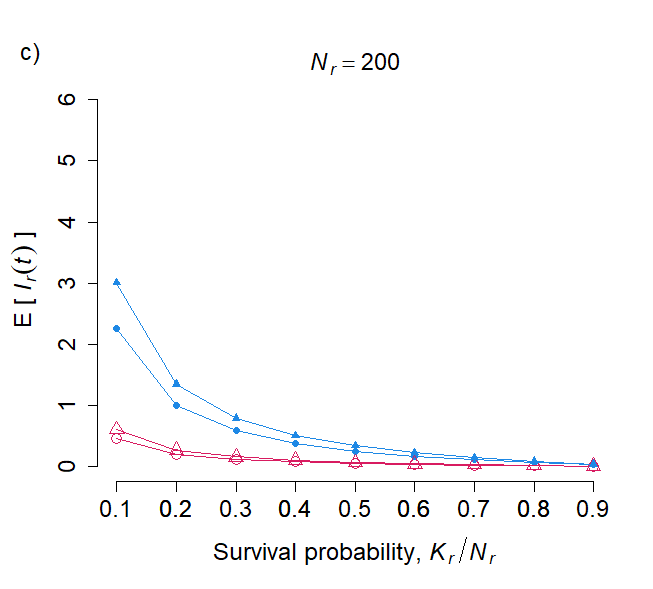

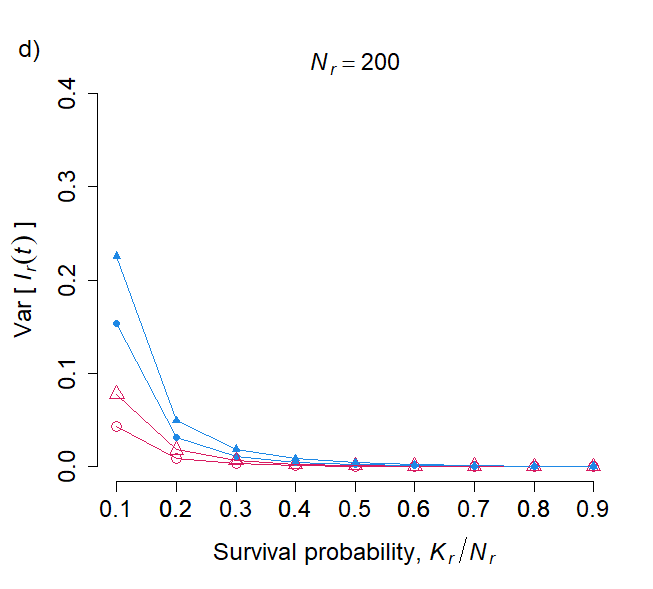


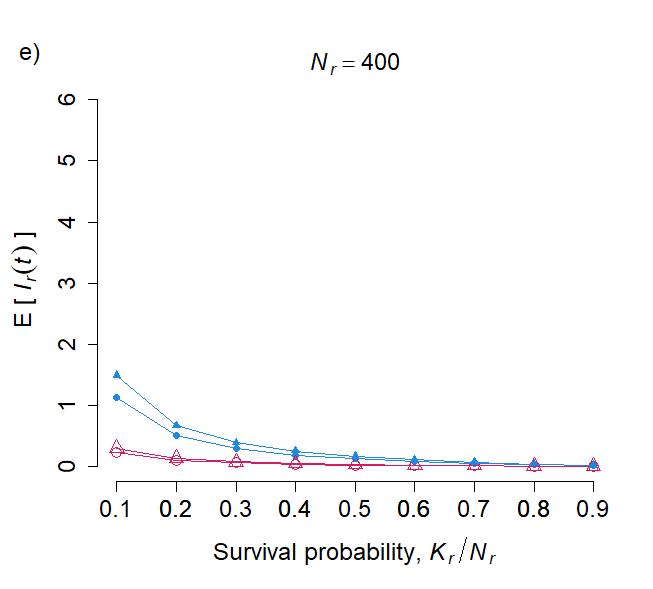

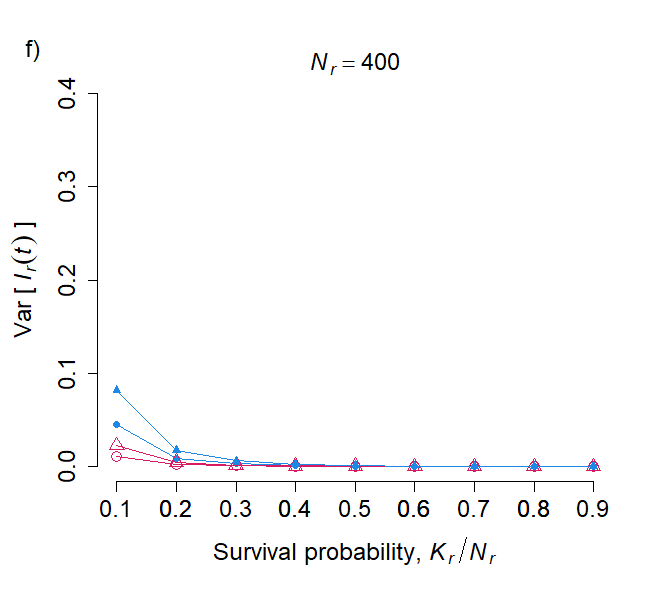


**Figure S6**. Length of the 95% confidence intervals of the null distribution of $I_{r}\left( t \right)$ for 108 combinations of the levels of four variables: three values of $N_{r}$ (100, 200, 400), two values of $n_{r}$ (10, 50), two levels of evenness in the distribution of plants among maternal lines (even and uneven). When the distribution of plants among maternal lines was even, all maternal lines had an initial number of plants $N_{ri}\left( 0 \right)=\frac{N_{r}}{n_{r}}$. In contrast, when the distribution of plants among maternal lines was uneven, half of the maternal lines had $N_{ri}\left( 0 \right)=\frac{1.5\times N_{r}}{n_{r}}$ and the other half $N_{ri}\left( 0 \right)=\frac{0.5\times N_{r}}{n_{r}}$. Each point (showing the length of the 95% confidence intervals of the null distribution of $I_{r}\left( t \right)$) was based on 10,000 iterations of the null model.


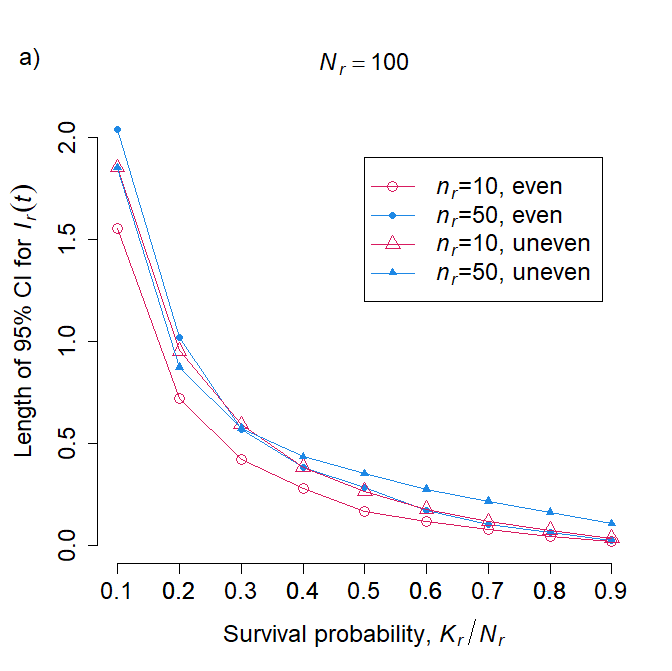


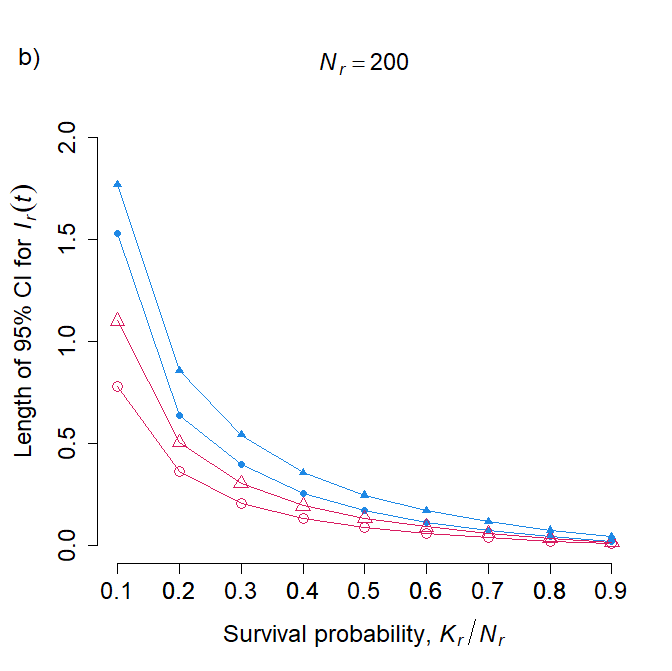


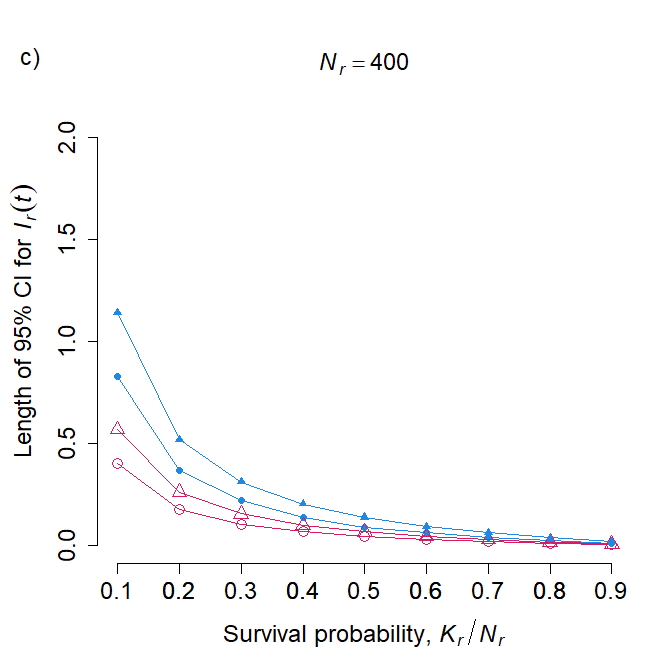

Supplement: Supplementary file 1 — Supporting Information [file COBI-40-e70350-s001.docx]
