# Supplementary material for: Measuring maternal line selection driven by differential survival in ex situ collections for plant conservation
Source: Conserv Biol. 2026 Jul 3;40(4):e70350. doi: 10.1111/cobi.70350 (PMC13392728; doi:10.1111/cobi.70350)

**Appendix S2: Spatial autocorrelation in individual plant survival**

In this appendix, we examined whether there was significant spatial autocorrelation in plant survival in the hoop house, as would be expected if the location of plants had a significant effect on survival due to environmental variation within the hoop house. Although we did not record the spatial coordinates of individual plants in the hoop house, we did record the number that was randomly assigned to each plant, independently from maternal line or provenance region, to determine their sequential position in the hoop house. We used this random number as an indication of their position in the hoop house in the analysis of spatial autocorrelation in individual plant survival. The R code for the analyses described in this appendix is available at: <https://figshare.com/projects/Measuring_selection_due_to_attrition_in_ex-situ_collections_for_plant_conservation/250889>

We first conducted a test of spatial autocorrelation (Legendre & Legendre 2012) in individual plant survival across spatial lags in plant position ranging from 1 to 30, indicating increasing separation between plants. For example, spatial lag 1 means that the correlation is estimated across plants that had neighboring positions in the hoop house, spatial lag 2 means that the plants were separated by one intermediate plant and spatial lag 3 means that the plants were separated by two intermediate plants. We constructed spatial autocorrelation functions that show the correlation coefficients for each spatial lag (Legendre & Legendre 2012). We assessed the statistical significance of correlations for each lag based on 5,000 iterations of a null model that randomized the location of individual plants while preserving survival data (whether the plant survived or not). The results indicated no statistically significant spatial autocorrelation in the survival data (Fig. S7a).

Second, we conducted a test of spatial autocorrelation in individual plant survival, after accounting for mean maternal line survival rate, across spatial lags in plant position ranging from 1 to 30. This second test sought to determine if individual deviations from mean maternal survival rates could be related to the location of plants in the hoop house. We carried out this test by calculating the spatial autocorrelation in the deviance residuals from the logistic regression of individual plant survival on maternal line. We used the “white noise” model (Box & Jenkins 2015) to assess the statistical significance of each lag in the spatial autocorrelation function. The results indicated no statistically significant spatial autocorrelation in individual plant survival, after accounting for mean maternal line survival rate (Fig. S7b).

**References**

Box, G. E. P., Jenkins, G. M., Reinsel, G. C., & Ljung, G. M. (2015). Time Series Analysis: Forecasting and Control (5th ed.). Wiley.

Legendre, P., & Legendre, L. (2012). Numerical Ecology (3rd English ed.). Elsevier.

**Figure S7.** Spatial autocorrelation function for a) individual plant survival and b) deviance residuals that estimate deviations of individual plant survival from mean maternal line survival rate. The horizontal axis shows spatial lags of plant position in the hoop house, from 0 to 30, indicating increasing separation between plants. Dots connected by the solid line show the autocorrelation coefficient (vertical axis) for each spatial lag. The autocorrelation coefficient for lag zero is (by definition) equal to 1, because it represents the correlation of each survival value with itself. Gray dashed lines show 95% confidence intervals for the null model of no correlation, calculated based on randomization of individual survival data (panel a) and the “white noise” model for deviance residuals (panel b).


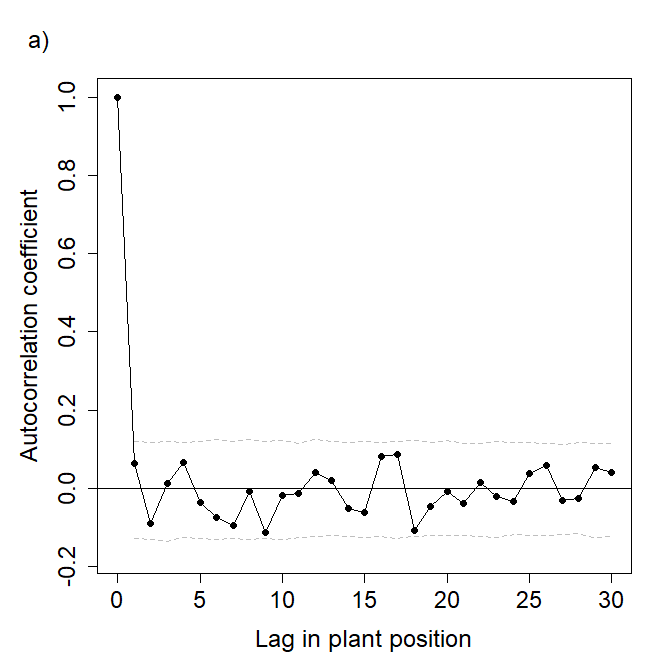


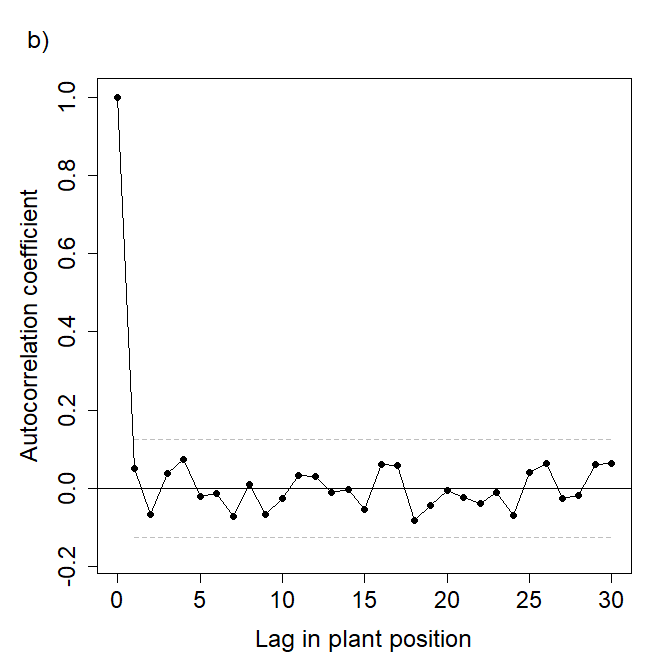

Supplement: Supplementary file 2 — Supporting Information [file COBI-40-e70350-s002.docx]
